# A study on the structural relationship between emotional labor, job burnout, and turnover intention among office workers in Korea: the moderated mediating effect of leader-member exchange

**DOI:** 10.1186/s40359-024-01545-8

**Published:** 2024-01-29

**Authors:** Yiran Li, Hyunok You, Seokyoung Oh

**Affiliations:** Department of Education, Yonsei University, 50, Yonsei-ro, Seodaemun-gu, Seoul, 03722 Republic of Korea

**Keywords:** Deep acting, Surface acting, Korean employees, Small- and medium-sized enterprises

## Abstract

**Background:**

This research investigated the interplay of emotional labor, job burnout, and leader-member exchange on turnover intentions among office workers in South Korea.

**Methods:**

An online survey was conducted with 333 employees working in Korean small- and medium-sized enterprises. The target sample consisted of in-house employees who do not deal with external customers. All the measurement and structural models of this study were analyzed using SPSS 27.0 and Amos 28.0.

**Results:**

The survey revealed that emotional labor indirectly influenced turnover intentions via job burnout and leader-member exchange. Deep acting intensified job burnout, thereby elevating turnover intentions, while surface acting mitigated job burnout.

**Conclusions:**

The findings underscored the importance of managing emotional labor and job burnout and fostering robust leader-member relationships to reduce staff turnover. Moreover, leader-member exchange was found to mitigate the effects of emotional labor on job burnout and turnover intention, with higher leader-member exchange reducing the negative impact of deep acting on turnover intention through job burnout.

**Supplementary Information:**

The online version contains supplementary material available at 10.1186/s40359-024-01545-8.

## Background

Emotional labor (EL) refers to the intentional control of emotions to adhere to organizational norms and display rules [1, 2]. Emotional workers, who are required to display specific emotions in their work, frequently experience emotional exhaustion and depersonalization [3]. Surface acting and deep acting are two types of EL that have both positive and negative outcomes [4]. While surface acting can create a positive work environment, it can also lead to feelings of inauthenticity and mental and emotional demands [5]. In contrast, deep acting can lead to job satisfaction, but may also result in emotional exhaustion if the job’s emotional demands are too high [6]. Despite performing professional, desk, or administrative work, office workers in South Korea can also be regarded as emotional laborers owing to the collectivistic nature of the organizational culture [7].

Drawing from the Affective Events Theory (AET), one can postulate that distinct emotional strategies (surface acting and deep acting) set the stage for varied affective responses in employees, which in turn mediate their reactions to work events. Specifically, as surface acting can lead to feelings of inauthenticity, it might precipitate negative affective reactions, potentially escalating turnover intentions (TIs) through the mediation of job burnout (JB). While initially fostering genuine emotional resonance and positive affective responses, deep acting can induce emotional exhaustion when one is overtaxed, further exacerbating turnover inclinations.

Crucially, the role of leader-member exchange (LMX) emerges as a potential moderating force in this nexus. Within an AET framework, high-quality LMX can serve as a buffer, ameliorating the negative affective reactions stemming from EL. For instance, when employees engage in surface acting, a robust LMX might validate their efforts, mitigating feelings of inauthenticity. Conversely, in the realm of deep acting, supportive leader-member dynamics might provide the requisite emotional scaffolding, reducing the propensity for emotional exhaustion despite the rigors of deep emotional modulation.

In summation, through the lens of AET, this study sought to elucidate the intricate interplay between the strategies of EL (surface and deep acting), their consequent affective reactions, and the modulatory influence of LMX, all culminating in determining TIs mediated by JB.

Per AET, the differential impacts of surface and deep acting in the realm of EL on TIs, mediated by job burnout, become evident. Surface acting, characterized by a display of emotions without genuine internal alignment, might impose a lesser emotional toll on workers compared to deep acting, which demands sincere emotional consonance. The absence of authenticity in surface acting paradoxically becomes its strength in this context. Since surface acting does not engage with genuine emotional depths, it results in reduced emotional expenditure. Consequently, this could lead to a diminished experience of JB, thereby potentially mitigating the inclination toward turnover. Conversely, the emotional consumption inherent to deep acting, demanding a true alignment of inner feelings with external displays, can be more taxing, potentially exacerbating job exhaustion and subsequent TIs.

### Literature review

Previous research has evaluated the two types of EL as responses to display rules. Both forms have been positively related to depersonalization or emotional exhaustion [3], and have negatively influenced job satisfaction [4]. However, empirical findings have been mixed and it has been found that both surface acting and deep acting have advantages and disadvantages. While surface acting can lead to a positive work environment, it may also lead to feelings of inauthenticity and mental and emotional demands [5]. Allen, Diefendorff, and Ma [8] argued that surface acting might elicit a positive response from Chinese employees as it helps maintain harmony and behavior according to social norms. Although deep acting can lead to a sense of authenticity with increasing job satisfaction [9], it can also lead to emotional exhaustion if the job’s emotional demands are too high [6]. Mastracci and Adams [10] emphasized that EL models proposed in the Western culture have limitations in explaining the collectivistic cultural contexts, and strategies may vary depending on cultural backgrounds. Few studies have revealed the characteristics of office workers’ EL, particularly in non-Western cultural contexts.

Recent studies have revealed that employees perceive JB as a job stress factor negatively affecting variables related to organizational effectiveness, such as job engagement, organizational commitment, or TIs [11–14]. Workers in their 30−40 s, who constitute the majority of the workforce, show a rapid increase in turnover rates owing to the emotional dissonance demanded by organizations.

This study focused on the role of LMX in the relationship between JB and TI. Previous studies have emphasized that EL and JB are reduced by horizontal LMX rather than vertical relationships within an organization [15, 16]. LMX may reinforce employees’ intrinsic motivation, psychological empowerment, organizational commitment, and organizational learning [17–21]. The study by Wu, Yuan, Yen, & Yeh [22] also showed that emotional support, such as inspiration and caring, mitigates burnout in situations with high emotional support and low job demand/control. Additionally, it was found that instrumental support, such as problem-solving, also alleviates the negative impact of burnout. Thus, we posited LMX as a variable moderating the relationship between JB and TI, analyzing the moderated mediation effect of LMX in the influence of EL on TI mediated by JB.

The purpose of this study was threefold. First, we identified how surface acting and deep acting EL affect JB in South Korean office workers, expanding research beyond office workers and exploring their relationships in a non-Western context. Second, we examined the mediation effects of JB in the relationship between EL and TI. Finally, we investigated whether LMX moderated the relationship between EL, JB, and TI. We posited that EL alone is not sufficient for TI and that LMX can mitigate the negative impact of EL and burnout on TI. By amalgamating the intricacies of EL, LMX, and TI, this scholarly endeavor presents an augmented discernment of catalysts propelling employee attrition, particularly in a milieu involving emotional exigencies. This study has important implications for elucidating the potential of superior leader-subordinate rapport in alleviating the burdens of EL, thus reducing employment termination.

### Theoretical background and hypotheses

#### Emotional labor, job burnout, and turnover intention

This study examined the moderating effect of LMX on the relationship between JB and TI. Prior research has suggested that horizontal LMX reduces EL and JB more effectively than vertical relationships [5, 16, 23], potentially reinforcing employees’ motivation, empowerment, commitment, and learning [20, 21]. Our study focused on how LMX moderates the effect of EL on TI, mediated by JB.

Investigations into the effects of EL have produced mixed results. Some studies have found that deep acting positively influences workers’ attitudes and behaviors by enhancing work efficiency and job satisfaction through the understanding of required display rules [24–27]. However, other studies have found no relationship between deep acting and emotional exhaustion or have suggested that it increases emotional exhaustion [28] and JB [3]. Despite the fact that deep acting is generally found to be less stressful and has fewer negative outcomes than surface acting, there is no consistent evidence to support its positive impact on employee outcomes, particularly JB. also, Güler, Okak, & Köksal [29] revealed that the relationship between natural emotional labor and organizational commitment was controlled by surface acting, but not by deep acting. It can be inferred that this is because if you choose to consume emotional resources with surface acting, you can increase organizational commitment, but if you choose to consume with deep acting, you can increase psychological exhaustion. In this regard, Chang, Chung, Chen, Lin, & Chen [30] found that stronger emotional support, such as servant leadership, can lead to employees having intrinsic motivation, such as psychological empowerment, to engage in deep acting to complete the performance expected by the organisation and leader, which in turn can increase psychological burnout. This is because deep acting can lead to psychological burnout in less capable members as they strive to deliver the results expected by the organisation and their superiors.

Similarly, studies examining the relationships between surface acting and employee outcomes have also produced mixed results. Surface acting, which involves suppressing true emotions and displaying organizationally desired responses, has often been described as harmful to employees [31]. However, in collectivist cultures like China and Korea, where strong interpersonal ties and harmony are emphasized, surface acting may be viewed as promoting positive responses and aligning with social norms [8]. Several studies have reported that surface acting has a lesser impact on burnout (such as personal accomplishment and emotional exhaustion) in Eastern cultures than in Western ones. Allen, Diefendorff, and Ma [8] found that employees in China experienced less emotional exhaustion from surface acting compared to employees in the US. Additionally, Mastracci and Adams [10] found that EL was less stressful and that the expression of surface acting was twice as common among people in collectivist cultures.

There is limited research on the EL of office workers who have few opportunities to interact with external customers. While some studies on EL among office workers have explored internal relationships where employees endure aggression or abuse from empowered individuals [32–34], we investigated EL in Korea, an Eastern collectivist culture, wherein previous research on EL has primarily been focused on Western cultures. Emotional regulation may be a normative process in collectivistic cultures, being regarded as necessary to maintain group harmony and cooperation [35]. While individualistic cultures tend to promote emotional expression, collectivistic cultures encourage emotional restraint and adaptation [36–39].

Deep acting demands an intrinsic modulation of one’s genuine emotions to align with organizational expectations. This requires a deeper introspection and alteration of one’s true feelings, leading to a potential emotional dissonance. This emotional dissonance, or the gap between one’s genuine feelings and the emotions they are expected to display, can be a source of significant stress. Deep acting is thus linked to an increase in JB. The consistent effort to regulate and modulate genuine emotions to meet external standards consumes significant emotional and psychological resources. This consistent EL over time can culminate in heightened burnout. Surface acting entails exhibiting emotions without necessarily feeling them internally. As it does not demand genuine emotional alignment, it often requires less psychological resource expenditure than deep acting. However, consistently masking one’s true emotions can still take its toll, leading to feelings of inauthenticity. Surface acting may reduce the immediate strain on psychological resources because it avoids the deep emotional processing and modulation that deep acting demands. By merely exhibiting the required emotions superficially, individuals can conserve the deeper, emotional reserves that deep acting might deplete. Surface acting gives the sensation of “not being on the job,” thus acting as a protective buffer against burnout by minimizing the consumption of psychological resources.

Deep acting, which demands an alignment of genuine emotions with organizational expectations, can increase JB due to the emotional dissonance it creates. Conversely, surface acting, which involves only the external display of emotions, might offer a temporary respite from emotional exhaustion because it avoids in-depth emotional processing. However, it is worth noting that both forms of EL, if not managed appropriately, can contribute to negative work outcomes in the long run.

Consequently, surface acting is often taken for granted in the workplace while deep acting is considered more stressful. Studies have reported that high levels of surface acting are associated with reduced fatigue and increased job satisfaction [11]. Surface acting can mitigate JB by minimizing the consumption of psychological resources. According to the research findings of Chen, Chang, & Wang [11], members who choose deep acting, when confronted with emotional labor, expend more emotional energy in order to achieve harmony between themselves and their job, while those who choose surface acting protect themselves from the reduction of emotional consumption and can alleviate exhaustion. Humphrey [40], who conducted a meta-analysis on burnout, acknowledged that while there is academic consensus that members engage in emotional labor in a workplace environment, the impact of emotional labor on members’ work behavior outcomes remains uncertain. Moreover, it was explained that deep acting leads to an increase in psychological burnout, such as emotional fatigue. Mastracci & Adams [41], in their study targeting public servants from different cultural backgrounds, explained that in individualistic cultures based on North American samples, display rules mediated by surface acting lead to an increase in burnout, and did not increase burnout when mediated by deep acting. However, in collectivist cultures based on Asian samples, display rules mediated by surface acting were found to increase burnout, and when mediated by deep acting, actually decreased burnout. This is attributed to public servants in collectivist cultures being more responsive to display rules compared to those in individualistic cultures. In their study focusing on members from the U.S. and Turkey, Nixon, Ceylan, Nelson, & Alabak [42] found that in the American sample, collectivism mitigated the impact of surface acting on emotional tension, a result not observed in the Turkish sample. Based on these differences, it can be posited that deep acting, involving significant emotional expenditure to achieve performance mandated by the organization and superiors, may ultimately lead to an inability to endure stress, thereby increasing burnout and turnover. Conversely, surface acting, chosen for its lower emotional expenditure, could potentially reduce burnout and turnover. This inference aligns with the nuanced complexities of emotional labor and its varied effects on employee well-being and organizational outcomes. However, it is important to note that emotional demands, whether involving fake or authentic emotions, can decrease job satisfaction and increase one’s intention to leave the organization [9]. Deep acting significantly affects JB [43–45]. Based on previous research and the Korean work context, we hypothesized as follows:

##### H1

Deep acting is positively related to TI.

##### H2

Deep acting is positively related to JB.

##### H3

Surface acting is negatively related to TI.

##### H4

Surface acting is negatively related to JB.

### Job burnout: a facilitator of turnover intention

JB is widely viewed as a facilitator of TI. Studies have shown that EL caused by conflict and stress increases employees’ JB, resulting in lower job motivation, satisfaction, and engagement [46–49]. Positive exchanges with the organization, superiors, and coworkers have been found to reduce employees’ EL, lowering JB and TI [50]. However, deep and surface acting increases without such exchanges, discouraging employees from performing duties for positive feedback and increasing their TI [11].

Organizations that emphasize high-performance work systems and innovation performance can exacerbate EL and psychological burnout [51, 52]. Employees experiencing higher EL may perceive less support from their organization and superiors, leading to psychological deprivation and a feeling of being unfit for the organization [53, 54].

Negative perceptions of EL can cause psychological problems, lower future organizational socialization, and result in JB and TI [55]. Employees who suppress negative emotions and attitudes to comply with an organization’s emotional display rules may experience higher JB, deviant behavior, and TI [56, 57]. The relationship between EL and TI can be explained through the AET [58]. AET outlines the causes and consequences of emotional labor, describing how the emotional states resulting from situational factors or job events perceived by individuals in the workplace shape their attitudes and behaviors (cognition → emotion → attitude → behavior). Previous studies on AET have been conducted, particularly targeting the general workforce, as it provides a useful framework for explaining the outcomes of events perceived by individuals in the workplace. This theory is useful for our study as employees’ affective reactions to job events have been shown to determine their EL [54, 55, 59, 60]. From this perspective, negative emotions and attitudes induced by EL can lead to TI, and both surface and deep acting can increase them. However, deep acting has a stronger effect [61]. The impact of EL on TI can also vary depending on individual differences and how employees interact with superiors and co-workers. Therefore, we also made the following hypotheses:

#### H5

Deep acting affects TI mediated by JB.

#### H6

Surface acting affects TI mediated by JB.

### Leader-member exchange: a moderator of turnover intention

In the present study, the moderating effect of employees’ perception of LMX on TI was examined. The effects of deep and surface acting on TI may vary depending on the perceptions of LMX and different mediation effects on JB. Further, it has been found that positive LMX can lower TI [62, 63]. Graen and Uhl-Bien [63] presented three stages of LMX: role taking, role making, and routinization. The three stages of LMX offer pivotal insights into mitigating the repercussions of both deep and surface acting on TIs via JB. In the role-taking phase, a nurturing leader can diminish the emotional strain from surface acting, preventing early burnout tendencies. As members transition to the role-making stage, with a more profound reliance on deep acting, empathetic leaders can detect and alleviate the emotional toll, countering the burnout that might otherwise fuel turnover aspirations. By the routinization phase, the deep-rooted bond between leaders and members acts as a consistent buffer against the negative facets of EL, ensuring that neither form culminates in overwhelming burnout or consequent TIs. If LMX between employees and superiors is carried out in that order, employees form in-groups where superiors offer kindness and support, which motivates employees to perform their duties more effectively. Consequently, this leads to a reduction in EL and mitigates the mediation effect of JB [64–67].

According to the social exchange theory, positive exchanges between superiors and employees lead to specific behaviors, work collaborations, and organizational learning, resulting in positive behaviors such as organizational commitment [68]. It is expected that positive LMX with superiors fosters pro-organizational behavior, mitigates the effects of deep and surface acting on TI, and increases the mediation effect in which deep and surface acting reduce TI mediated by JB [69–72]. This is because employees tend to reduce their TI if they perceive high LMX with superiors [63].

However, if LMX with superiors is perceived as low, it will fail to reduce TI [73]. Without clear LMX with superiors, employees may not feel dutiful toward the organization. Harris, Li, and Kirkman [74] have analyzed the relationship between superiors’ LMX and TI and confirmed that TI peaks when LMX is low, whereas higher LMX leads to lower TI. This relationship varies depending on the level of positive LMX between superiors and employees. If employees have a low level of JB, they tend to perceive the provided LMX higher. Therefore, reducing the JB of employees through superior-employee interactions is essential. As employees pass the three stages of role taking, role making, and routinization, role ambiguity, and TI are minimized, and receptivity toward LMX with superiors is increased. The study by Wu, Yuan, & Yen [75] also revealed that turnover intentions decreased in the group with high leader-member exchange (LMX), as the exchange relationships between bosses and colleagues were stronger in this group compared to the group with low LMX.

**Fig. 1:**
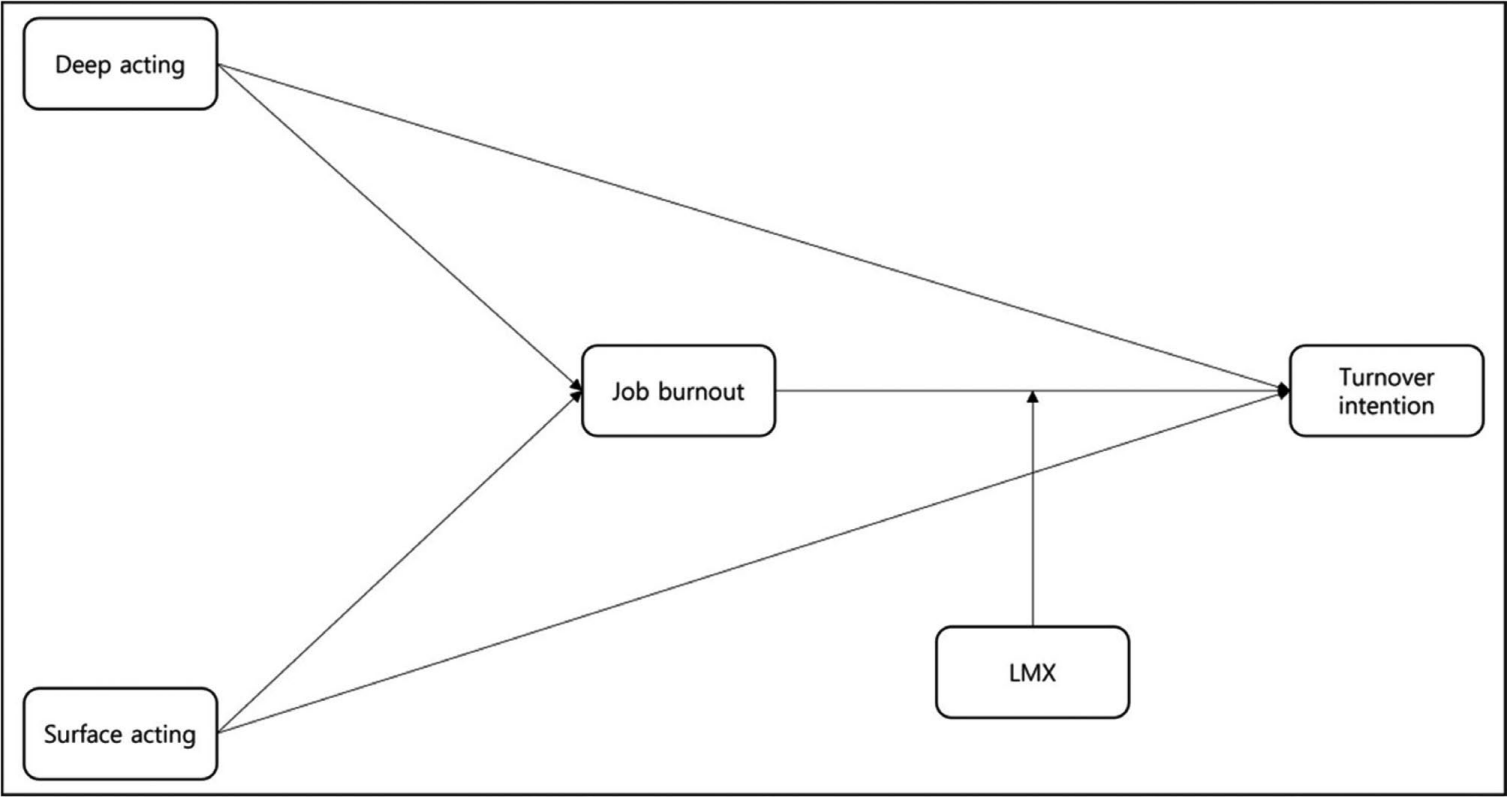
Study model

Similarly, Chen et al. [76] revealed that employees’ emotions and attitudes toward their superiors were essential for reducing TI; employees with high fellowship had lower JB and TI. High fellowship refers to the profound sense of camaraderie and mutual respect between employees and their superiors, which, as Chen et al. have highlighted, plays a pivotal role in diminishing JB and TIs by fostering positive emotions and attitudes toward leadership [Fig. 1]. Therefore, the following hypotheses were also made:

#### H7

LMX mitigates the effect of deep acting on TI, mediated by JB.

#### H8

LMX mitigates the effect of surface acting on TI, mediated by JB.

## Methods

### Participants

This study examined the effect of EL (deep acting, surface acting) on TI, mediated by JB, and empirically analyzed the moderated mediation effect of LMX. We surveyed employees working in Korean small- and medium-sized enterprises (SMEs). The target sample consisted of in-house employees who do not deal with external customers. To increase the participation of employees who are potentially engaged in high EL, the consent form stipulated that this study aimed to identify burnout and TI owing to EL. This study conducted an online survey on Survey Monkey, distributed a questionnaire from 15 July to 15 December 2019, and received 450 responses. We chose SurveyMonkey for its efficiency, accessibility, and familiarity to respondents, ensuring higher response rates and a seamless participant experience, despite concerns regarding its academic validation in scholarly research. Furthermore, 117 responses were excluded because of insincere responses, leaving 333 valid samples. By “insincere responses,” we allude to those responses that contained absent or incongruent data, suggesting an absence of earnest engagement or meticulous attention throughout the survey undertaking. To use G*Power 3 to analyze five measurement variables, two independent variables, and one mediator variable, we needed to collect at least 138 samples [77].

Among the participants, 233 were male (70%). By age group, 78 participants were in their 20s (23.4%), 138 in their 30s (41.4%), 71 in their 40s (21.3%), and 46 were aged 50 or older (13.8%). Regarding education level, 75 participants were high school graduates or lower (22.5%), 244 were college graduates (73.34%), and 14 were graduate school graduates (4.2%). With respect to work positions, 152 participants were staff-level employees (45.6%), 106 were assistant managers (31.8%), 42 were managers (12.6%), and 33 were senior managers (9.9). Most had worked with their superiors for one to three years (217, 65.2%) and 185 had worked for one to five years (55.6%) in their current firm. Lastly, 289 employees (86.8%) were tenured.

### Measurement tools and analysis method

#### Measurement tools

##### Emotional labor

Six items were used to measure emotional labor for both surface acting (e.g., employees try to change their feelings to align with the emotions required by the organization) and deep acting (e.g., employees express themselves according to the display rules and norms required at work while hiding their true emotions) as developed by Diefendorff, Croyle, and Gosserand [64]. rated on a 5-point Likert scale (1 = Strongly disagree, 5 = Strongly agree). Drawing from prior research, the validity of this scale has been confirmed through confirmatory factor analysis on EL (higher than 0.600), with reliabilities of 0.820 and 0.850 for deep acting and surface acting, respectively [64].

#### Job burnout

The Maslach Burnout Inventory (MBI) [78], adapted and validated by Kang (1996) [79], was used to measure JB. We obtained a licence to use this scale from authors and used the Korean-validated MBI from Kang Hak Koo’s (1996) licensed research. The MBI comprises nine items: five items on exhaustion (e.g., employees feel emotionally exhausted doing their job) and four on cynicism (e.g., employees passively do their job). Rated on a 7-point Likert scale (0 = Never, 6 = Every day). The validity of the scale was demonstrated in previous studies with factor loadings of exhaustion and cynicism between 0.083 and 0.087 [80, 81]. Kang [79] proved scale reliability with exhaustion = 0.857 and cynicism = 0.639. In this study, the MBI (factor loading 0.673−0.822) was measured to a level that can accept latent variables, and the total reliability was 0.931. The subscales of exhaustion and cynicism had reliabilities of 0.900 and 0.896, respectively. Therefore, JB was measured with a second-order factor model, as it has a second-order factor structure of exhaustion and cynicism. In this study, the Maslach Burnout Inventory (MBI) was utilized as per the guidance of its author, and the tool was officially licensed through Mind Garden, Inc. (www.mindgarden.com).

#### Turnover intention

The Turnover Intention Scale by Landau and Hammer [82] was used to measure employees’ TI. This scale comprises three items (e.g. actively searching for a new job) rated on a 5-point Likert scale (1 = Strongly disagree, 5 = Strongly agree), with higher scores indicating greater TI. The scale was previously measured with a single factor, and the factor loading was valid at 0.500 or higher [82]. Furthermore, the reliability was 0.900 in Landau and Hammer’s [82] study.

#### LMX

The tool to measure LMX, developed by Graen and Uhl-Bien [63], was translated for this study, and the seven items were revised to suit the context (e.g., awareness of superior’s satisfaction with work performance). rated on a 5-point Likert scale (1 = Strongly disagree, 5 = Strongly agree). The LMX scale was previously measured with a single factor, and the factor loading was valid at 0.500 or higher [63]. Additionally, the reliability exceeded 0.700.

### Data analysis

The measurement and structural models of this study were analyzed using SPSS 27.0 and Amos 28.0. Reliability and confirmatory factor analysis were conducted to secure internal consistency and content validity based on operational definitions and measurement tools from previous studies. Harman’s One Factor Test and post hoc tests were used to verify the common method bias. Correlations and discriminant validity were analyzed, and structural model analysis was conducted to test the hypotheses. The moderated mediation effect was explored using Process Macro, which uses a partial least squares algorithm to find the trend line that rises most linearly in linear regression analysis [83]. The primary aim was to explore the mediation effect of deep acting and surface acting on EL, JB, and TI, and the moderated mediation effect of LMX. The hypotheses were also tested using Amos.

## Results

### Measurement model

Reliability was checked using Cronbach’s Alpha values (CAV) before testing the measurement model. Internal consistency was found among items for the six items on deep acting and six on surface acting (α = 0.856 and 0.871, respectively), five items on exhaustion and four on cynicism with JB as a mediator (α = 0.900 and 0.896, respectively), three TI items (α = 0.892), and seven LMX items (α = 0.901) [84]. The CAV of each variable’s items ranged from 0.856 to 0.931, indicating high reliability. Common method bias was tested by using Harman’s One Factor Test. It showed that the variance for one factor did not exceed half of the total variance of all the constructs (31.12%), which means that the variance on any one factor did not exceed half of the total variance of all components and that common method bias is not a significant problem [85]. Confirmatory factor analysis showed that the analytical model setting of all variables as one latent factor had a significantly lower fit than the measurement model ($$ {x}^{2}$$=3113.357, degrees of freedom; Df = 252, ratio of chi-square minimum and degrees of freedom; CMIN/DF = 12.355, goodness of fit index; GFI = 0.396, adjusted goodness of fit; AGFI = 0.465, comparative fit index; CFI = 0.396, normed fit index; NFI = 0.379, incremental fit index; IFI = 0.399, Tucker-Lewis index; TLI = 0.339, root mean square error of approximation; RMSEA = 0.185, root mean residual; RMR = 0.156), indicating that standard method bias did not affect the results [85].

The standardized coefficients of all Items on EL, JB, TI, and LMX were higher than 0.500, ensuring the construct reliability of all items [83]. Convergent validity was also ensured, as the standardized coefficients of survey items were higher than 0.500. The model fit of convergent validity analysis in Table 1 met the standard for the absolute fit index, incremental fit index, and parsimonious fit index of the structural equation model. The average variance extracted (AVE) and composite reliability (CR) for each construct’s reliability, convergent validity, and discriminant validity were also tested. Table 1 shows that all variables used in this study had an AVE higher than 0.500 and a CR higher than 0.800, meeting the two construct reliability requirements [83, 86].

**Table 1:** Reliability, validity, and confirmatory factor analysis results

Variable		Estimate	SE	CR	*P*	AVE	Composite reliability
Deep acting	DA 1	0.685	0.076	9.055	***	0.578	0.891
	DA 2	0.852	0.070	12.223	***		
	DA 3	0.838	0.066	12.741	***		
	DA 4	0.910	0.066	13.778	***		
	DA 5	1.000	-	-	-		
	DA 6	0.851	0.062	13.816	***		
Surface acting	SA 1	1.000	-	-	-	0.592	0.896
	SA 2	0.885	0.059	14.978	***		
	SA 3	0.845	0.065	13.027	***		
	SA 4	0.725	0.063	11.558	***		
	SA 5	0.911	0.064	14.161	***		
	SA 6	0.988	0.065	15.126	***		
Job burnout	Cynicism	1.000	-	-	-	0.876	0.934
	Burnout	0.883	0.055	16.19	***		
Turnover intention	TI 1	0.871	0.033	26.244	***	0.827	0.935
	TI 2	0.781	0.046	17.084	***		
	TI 3	1.000	-	-	-		
LMX	LMX 1	1.000	-	-	-	0.633	0.923
	LMX 2	1.023	0.047	21.616	***		
	LMX 3	0.862	0.057	15.079	***		
	LMX 4	0.820	0.064	12.736	***		
	LMX 5	0.796	0.049	16.194	***		
	LMX 6	0.849	0.051	16.524	***		
	LMX 7	0.588	0.057	10.370	***		

We analyzed the relationship between latent variables and assessed discriminant validity to ensure independence among variables (deep and surface acting as independent variables, JB as a mediator, TI as a dependent variable, and LMX as a moderator). Discriminant validity was examined by comparing the AVE of latent variables and the squares of correlation coefficients among them [86]. Table 2 shows that the AVE of latent variables is greater than the squares of correlation coefficients, indicating discriminant validity [86, 87]. Furthermore, discriminant validity was confirmed as all heterotrait-monotrait ratio of correlations (HTMT) values were below 0.850 (or 0.900) [88]. Based on the criteria, discriminant validity is generally confirmed when all HTMT values are below the threshold of 0.850 or, in more lenient cases, 0.900. In our study, we found that all HTMT values were indeed below this threshold, indicating that our measures exhibit sufficient discriminant validity.

**Table 2:** Correlation and discriminant validity analysis

Variable	M	S.D	Deep acting	Surface acting	Job burnout	Turnover intention	LMX
Deep acting	3.2863	0.65906	(0.578)				
Surface acting	3.3589	0.61695	0.134* H:0.186	(0.592)			
Job burnout	2.7695	0.83717	0.411** H:0.474	− 0.178** H:0.223	(0.876)		
Turnover intention	2.5305	1.14896	0.237** H:0.265	− 0.147** H:0.170	0.549** H:0.626	(0.827)	
LMX	3.3724	0.78878	− 0.176** H:0.197	0.378** H:0.410	− 0.469** H:0.510	− 0.326** H:0.350	(0.633)

**Notes**: Significant at the level of **p < 0.01, *p < 0.05; Bold numbers in brackets in the diagonal line are AVE; Numbers below the diagonal line are correlations between constructs; H values under the correlation coefficients are HTMT thresholds

### Structural model

**Fig. 2:**
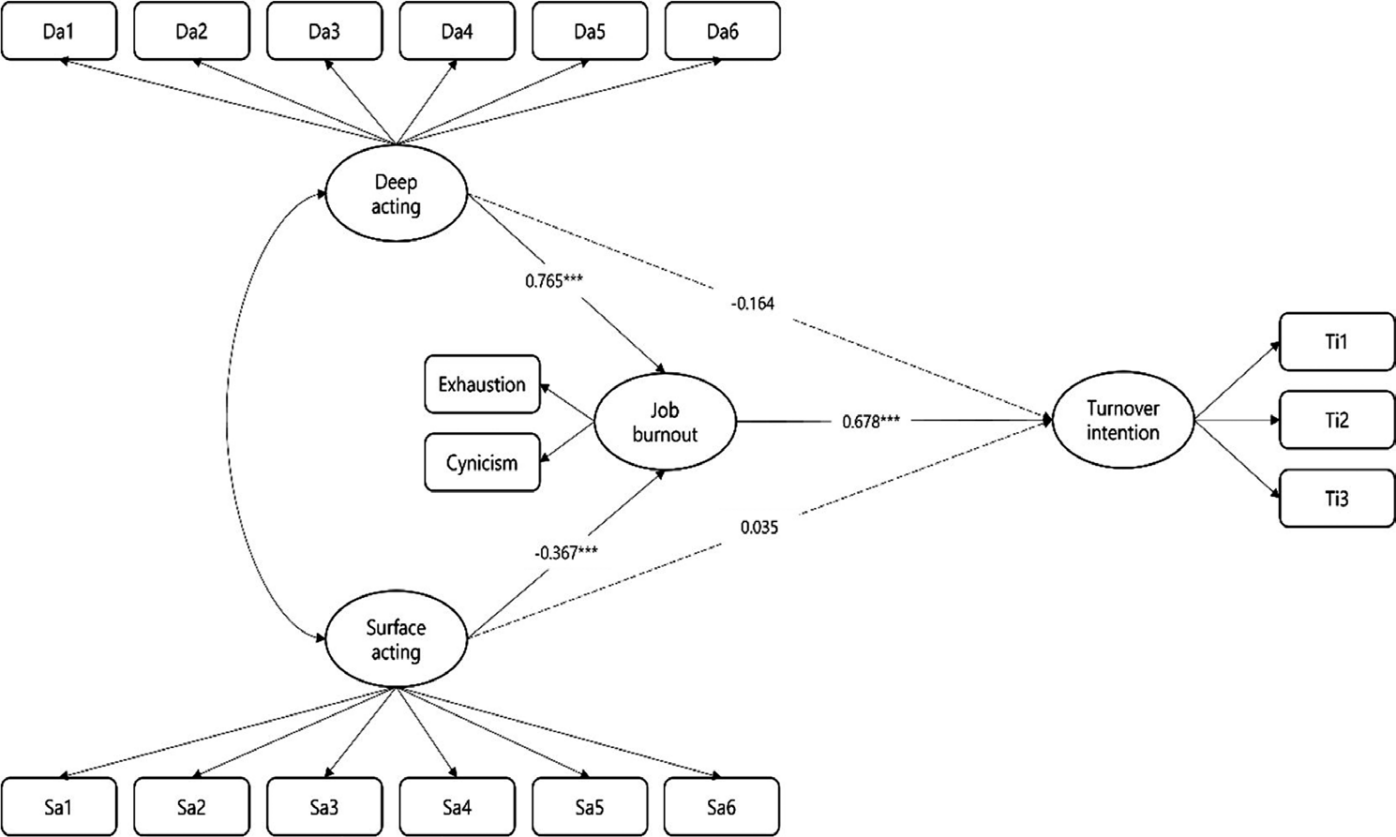
Structural model in which deep acting and surface acting affect turnover intention through job burnout

A structural equation model was used to test the hypotheses, controlling for gender, age, education level, work position, length of work experience, and employment status. Deep acting had a significant positive effect on JB (β = 0.765, *p* < 0.000), but not on TI (β=-0.164, *p* > 0.05), thereby rejecting Hypothesis 1 and accepting Hypothesis 2. Surface acting did not have a significant positive effect on TI (β = 0.035, *p* > 0.05), but had a significant negative effect on JB (β=-0.367, *p* < 0.000), thereby rejecting Hypothesis 3 and accepting Hypothesis 4. To test Hypotheses 5 and 6, 5,000 samples were extracted and estimated using a bootstrap bias-corrected method. Results confirmed that JB played a significant mediating role in the relationship between deep acting and TI (β=-0.072 direct effect, β = 0.304 indirect effect) and between surface acting and TI (β = 0.019 direct effect, β=-0.178 indirect effect), accepting Hypotheses 5 and 6. Hypothesis 7 was also supported, indicating that deep acting had a significant positive effect on TI mediated by JB, while surface acting had a significant negative effect on TI mediated by JB. Table 3 presents the structural model analysis results [Fig. 2].

**Table 3:** Structural model analysis results

Path			Estimate	SE	CR	P	Standardized coefficient
Gender	→	turnover intention	0.179	0.112	1.598	0.110	0.076
Age	→	turnover intention	0.007	0.066	0.108	0.914	0.006
Education	→	turnover intention	-0.108	0.056	-1.917	0.055	-0.088
Position	→	turnover intention	0.118	0.050	2.384	0.017	0.125
Work experience	→	turnover intention	-0.108	0.049	-2.218	0.027	-0.119
Employment status	→	turnover intention	-0.453	0.149	-3.047	0.002	-0.142
Gender	→	job burnout	-0.217	0.095	-2.287	0.022	-0.123
Age	→	job burnout	-0.061	0.056	-1.087	0.277	-0.073
Education	→	job burnout	0.036	0.048	0.739	0.460	0.039
Position	→	job burnout	-0.076	0.042	-1.806	0.071	-0.107
Length of service	→	job burnout	0.038	0.042	0.916	0.360	0.056
Deep acting	→	turnover intention	-0.164	0.134	-1.218	0.223	-0.072
Surface acting	→	turnover intention	0.035	0.096	0.369	0.712	0.019
Job burnout	→	turnover intention	0.901	0.095	9.515	***	0.678
Employment status	→	job burnout	0.136	0.127	1.076	0.282	0.057
Deep acting	→	job burnout	0.765	0.122	6.261	***	0.448
Surface acting	→	job burnout	-0.367	0.080	-4.604	***	-0.263
Job burnout	→	turnover intention	0.901	0.095	9.515	***	0.678
Mediation effect			Indirect effect	Direct effect	Total-effect	Lower bound	Upper bound
Deep acting → job burnout → turnover intention			0.304	-0.072	0.232	0.218	0.427
Surface acting → job burnout → turnover intention			-0.178	0.019	-0.159	-0.250	-0.106

### Moderated mediation effect

In this study, variables were mean-centered to minimize multicollinearity and avoid interpretation errors of moderating effects [89]. The moderated mediation model, which combines the moderator variable with the mediation model, was analyzed using Process Macro 4.0 Model 14 [83]. The results indicated that LMX moderated the indirect effect of deep acting on TI through JB (Table 4). The findings demonstrated that employees engaging more in EL tend to experience higher levels of JB, subsequently increasing their TI (mediation effect). However, this mediated relationship was less prominent among those with elevated LMX levels (moderating effect). Contrary to expectations, LMX did not moderate the indirect effect of deep acting and surface acting on TI through JB, indicating that this effect happens through JB alone. In addition, LMX did not mitigate the indirect effect of surface acting on TI through JB. Employees who engage more in EL experience reduced JB, and surface acting decreases JB and job stress, resulting in reduced TI. Conversely, deep acting induces JB and increases job stress, leading to an increase in TI. However, increasing LMX mitigates the indirect effect of deep acting on TI through JB. This is because LMX decreases the indirect effect of JB on TI, encouraging employees to remain with the organization. Consequently, the study reveals that LMX plays a moderating role in the relationship between deep acting and TI, mediated by JB. Specifically, while employees engaging in more EL tend to experience increased JB leading to heightened TI, this effect is less pronounced for those with higher LMX. Surprisingly, LMX does not influence the indirect effects of both deep and surface acting on TI via JB. While surface acting reduces JB and subsequently lowers TI, deep acting amplifies JB, thus increasing TI. Nevertheless, higher LMX can buffer the negative effects of deep acting on TI by reducing the impact of JB, thereby promoting employee retention.

**Table 4:** Indirect effect of job burnout on turnover intention according to LMX

Path		Index	BootSE	LLCI	ULCI
deep acting → job burnout → turnover intention		-0.0514	0.0321	-0.1074	-0.0008
Moderator variable	LMX	Effect	BootSE	95% confidence interval (minimum and maximum value)	
M-1SD	2.5836	0.4042	0.0751	0.2848	0.5313
M	3.3724	0.3636	0.0666	0.2582	0.4773
M + 1SD	4.1612	0.3231	0.0672	0.2198	0.4391

**Notes**: *n* = 333, significant when there is no 0 between the lower bound and upper bound; LMX, leader-member exchange; SE, standard error; LLCI, lower level of confidence interval; ULCI, the upper level of confidence interval

**Fig. 3:**
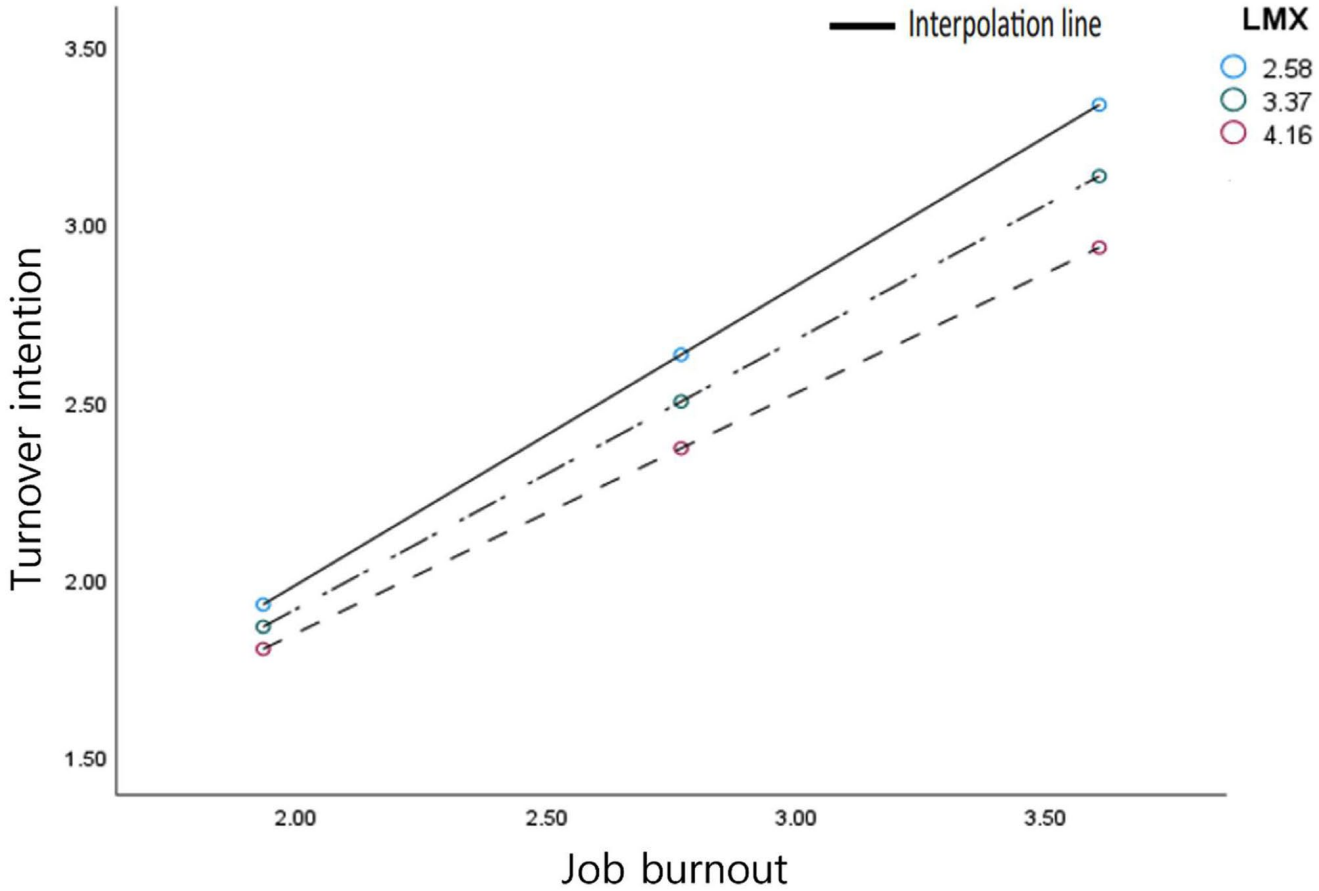
Relationship between job burnout and turnover intention according to leader–member exchange

Table 4; Fig. 3 indicate a significant moderated mediation effect of LMX on the relationship between deep acting and TI through JB. The 95% confidence interval showed a significant lower bound (-0.1074) and upper bound (-0.0008). When LMX was low, the mediation effect was β = 0.4042, whereas when it increased to medium and the highest point, the mediation effect weakened to β = 0.3636 and β = 0.3231, respectively, indicating a significant moderated mediation effect of LMX on the relationship between deep acting and TI through JB. However, the impact of surface acting on TI via JB did not show a similar attenuation based on LMX levels.

## Discussion

This study explored the factors affecting employees’ TI. Two actions of EL—surface acting and deep acting—JB and TI, were selected as predictor variables based on a literature review and AET. Moreover, LMX was also predicted to affect TI. This study proposed hypotheses regarding whether the two forms of EL affected TI through JB and tested them using structural equation modeling. Additionally, it proposed hypotheses on the mitigating effect of LMX on JB and TI and explored the moderated mediation effect.

First, neither deep acting nor surface acting had a significant effect on TI. TI does not increase regardless of whether employees act superficially or try to internalize the required actions. These findings contradict previous studies, which suggested that deep acting lowers TI among service workers [90–92] and surface acting has a positive effect on TI [93]. Second, deep acting increased JB, while surface acting decreased JB. This result also contradicts previous studies wherein deep acting reduced job exhaustion, whereas surface acting exacerbated it [24, 34, 94]. Unlike employees whose main tasks involve EL, employees with individual tasks use energy to internalize the required actions into sincere actions, presumably increasing JB. Contrarily, expressing superficially demanded behavior decreases JB regardless of real emotions. Additionally, aligned with Zhao, Li, and Shields’ [88] results, ours showed that the negative relationship in which EL affected JB became a positive relationship as EL increased. This may be because both positive and negative aspects exist simultaneously in EL. Though most previous studies have shown that inner behavior reduces job exhaustion and expression behavior increases job exhaustion, there is a high probability of different results owing to the opposite inner and superficial behavior of EL. This aligns with Yin, Huang, and Chen’s [95] meta-analysis.

Third, irrespective of whether JB mediated the effect of EL on TI, deep acting increased TI through JB. Sincerely matching an organization’s display rules causes JB, increasing TI. Previous studies of service workers have found that performing deep acting reduces JB and TI [34, 94, 96, 97], whereas research on non-service workers has yielded conflicting results. For instance, Grandey et al. [98] found that non-service workers experienced less emotional labor compared to their counterparts in service roles. Conversely, Diefendorff et al. [99] argued that the emotional demands on non-service workers could be equally intense, albeit manifested differently. Thus, in addition to performing one’s duties, more energy is spent sincerely matching the display rules required by the company. This finding complements previous studies that have emphasized that the effect of inner acts cannot be positive and that it is necessary to verify the complex effects of inner acts on JB [100]. Furthermore, this study is similar to Kammeyer-Mueller et al.’s [101] and Grandey, Rupp, and Brice’s [32], which demonstrated that deep acting and surface acting can have either positive or negative effects. Therefore, EL may vary depending on the work environment and organizational culture (collective or individualistic), and emotions owing to EL may vary depending on situational factors.

Fourth, LMX mitigated the effect of both deep acting and surface acting on TI mediated by JB; deep acting affects TI through JB, reducing TI more in the group with higher LMX than in the group with lower LMX. Significantly, the effect of EL on JB and TI varies depending on the level of LMX. Given the results showing how LMX moderates the effects of EL on TIs, the implications for collectivistic and individualistic cultures are notable.

In collectivistic cultures, where group harmony and interpersonal relationships are paramount, a higher LMX might be especially influential. These cultures emphasize the value of close, cooperative relationships, which can buffer the strains of emotional labor. The significant moderating effect of LMX in these cultures suggests that building strong leader-member relationships can be an effective strategy to reduce the adverse effects of deep acting and surface acting on TIs mediated by JB.

Conversely, in individualistic cultures, where personal autonomy and individual achievement are prized, the impact of LMX might be nuanced. While a positive leader-member relationship is beneficial, individuals might be more focused on their personal coping mechanisms to handle the demands of EL. Therefore, while LMX can still have a moderating effect, its influence might be less salient compared to collectivistic settings. However, in an individualistic culture, LMX can be the basis for supporting personal performance and enhancing innovative work behavior [102].

In essence, the cultural backdrop can shape how leader-member relationships interact with EL processes, underlining the importance of cultural considerations in organizational interventions and strategies. These results align with Wu and Hu’s [103] argument that the exchange relationship between managers and subordinates alleviates negative results when members engage in positive or negative EL. In this study, we examined how the impact of internal behavior on job exhaustion is moderated by LMX. In situations where emotional exhaustion is prevalent, we proposed that LMX—a situational factor—can allow team members to decrease their emotional expenditure. This mitigation effect occurs as higher levels of LMX can potentially reduce the emotional toll. Surface acting, being a type of EL where employees outwardly display emotions they do not genuinely feel, may be particularly draining and resistant to the buffering effects of strong leader-member relationships. While high LMX can reduce the emotional toll in many scenarios, the inherent nature of surface acting might make it uniquely challenging, suggesting that the superficiality and inauthenticity of surface acting may not be easily mitigated even in the presence of strong interpersonal ties with leaders. This underscores the complexity of emotional processes in the workplace and indicates that not all emotional labor components can be effectively buffered by strong leader-member dynamics.

### Theoretical and practical implications

First, this study reconfirmed the effect of EL on JB and TI, as found in human resources management studies. Drawing from Grandey’s [104] study on EL, our research reconfirmed the significant influence of EL on JB and TIs. These findings emphasize the importance for human resource managers to implement strategies that mitigate the effects of EL, ensuring employee well-being and retention. Particularly, in this context, this refers to our study’s reaffirmation of the link between EL, JB, and TIs, while also exploring the intricate relationships among these three elements. The results will help organizations develop and manage human resources in the future. The current study distinguishes itself from prior research by not only reaffirming the connection between EL, JB, and TIs but also delving deeper into the structural relationships among these three factors. While earlier studies have primarily focused on individual relationships between these variables, our research presents a comprehensive framework that captures their interdependencies. This enhanced understanding extends existing knowledge by providing a holistic view of how these factors interrelate, offering valuable insights for organizations. Specifically, by comprehending these intricate relationships, organizations can tailor intervention strategies to address the root causes of JB and turnover, ensuring a more effective approach to employee well-being and retention. We reveal the discriminatory effects of deep action and surface action of EL, suggesting that human resource development policies can be applied individually to members. The study’s findings underscore the crucial role of HR policies in addressing the challenges posed by emotional labor and its consequent effects on job burnout and turnover intentions. It is imperative for organizations to adopt proactive measures, including specialized training, regular employee monitoring, and supportive programs like mentorship. Additionally, providing flexible work arrangements and interventions for those displaying high turnover intentions can be pivotal. In essence, prioritizing employee well-being through tailored human resource strategies not only enhances individual productivity but also bolsters overall organizational stability and success.

Second, this study divided EL into deep acting and surface acting to reveal complex relationships. Considering that one hypothesis testing result supported the proposed effect of deep acting on TI and JB, it can be assumed that deep acting causes more stress and negative outcomes; mere surface-level acting has a relatively low impact on increasing JB and TI. Here, office employees are forced to engage in deep acting by human resources departments, which consider that truly relating to and caring for customers and the outward expression of this is the best form of service provision. However, for the workers in this study, surface acting rather than deep acting may improve their results. Additionally, along with the results of previous studies wherein there is ambivalence in EL, most of this study also derived results different from previous studies [104]. As those who partake in deep acting regularly perform office work, it causes emotional consumption. Surface acting does not increase emotional consumption, but suggests that JB can be reduced.

Third, while many previous studies have attached importance to the relationship between superiors and subordinates for TI, the significance of this linkage was not found when EL was involved. Although employees engaging more in deep acting had higher JB, which increased TI, LMX level mitigated this. Thus, to mitigate TI, it is essential to change LMX interventions and create an organizational atmosphere to manage deep and surface acting correctly. The findings suggest that while deep acting leads to higher levels of JB, surface acting does not necessarily increase emotional exhaustion in the same manner. In the context of the study, surface acting does not intensify emotional consumption to the same extent as deep acting. This distinction emphasizes the importance of differentiating between these two forms of EL and tailoring organizational strategies accordingly to manage the emotional well-being of employees. Moreover, from an AET perspective, the results suggest that applying LMX to members who perform deep acting can reduce the effect leading to TI.

### Limitations and suggestions for future research

Our study had a few limitations. This cross-sectional study used randomly selected samples and was unable to completely avoid standard method bias [105]. However, data collected through online surveys are less prone to exaggeration than other survey data. Online surveys, though cost-effective and convenient, can introduce biases due to self-selection, the digital divide, and misunderstandings of questions. They may also face issues from limited engagement, fraudulent responses, technical glitches, and the absence of non-verbal cues. It is crucial to consider these potential pitfalls when using online surveys. Harman’s single-factor test and post-hoc test were conducted to verify common method bias. Future studies could measure the variables longitudinally from various sources to reduce the bias. However, it is worth noting that the lack of control over variables such as compensation levels and job characteristics in the process of sample selection could introduce confounding factors that might engender spurious results. To address these potential biases, future studies could measure the variables longitudinally from various sources.

Additionally, the study participants were diverse, including employees from various industries, which limits the generalizability of the findings. Future studies could compare organizational cultures between SMEs and large enterprises, or between service and other industries, to address this limitation.

Further, this study only measured specific variables (predictor, mediator, dependent, and moderator) rather than running a time-series analysis or experimental research to establish causal relationships and provide solutions for EL. This limitation means that there could be inaccuracies in investigating the causal relationships and providing solutions for social phenomena. Collecting regional data could help alleviate this issue.

Lastly, the study used JB as the mediator variable and LMX as the variable that mitigates the mediation effect of JB. The effect of LMX as a contextual factor cannot be ignored even though JB plays a mediating role. An in-depth discussion on the importance of studying the relationships in Asian contexts is necessary, with a spotlight on the unique contributions related to non-service employees and the office context.

## Conclusions

This study examined the impact of EL on JB and TI among employees. The findings revealed that neither deep acting nor surface acting had a significant effect on TI, contrary to previous studies. Deep acting increased JB, while surface acting decreased JB, contradicting prior research. Moreover, LMX was found to mitigate the effects of EL on JB and TI, with higher LMX reducing the negative impact of deep acting on TI through JB. The study highlights the complex and situational nature of EL and its effects on employee outcomes.

### Electronic supplementary material

Below is the link to the electronic supplementary material.

Supplementary Material 1

## Data Availability

Data resources and statistical analysis outputs can be provided by the corresponding author (Yiran Li) on reasonable request.

## Abbreviations

EL: Emotional labor
AET: Affective Events Theory
LMX: Leader-Member Exchange
JB: Job burnout
TI: Turnover intention
SME: Small- and medium-sized enterprise
MBI: Maslach Burnout Inventory
CAV: Cronbach’s Alpha values
Df: Degrees of freedom
CMIN/DF: Ratio of chi-square minimum and degrees of freedom
GFI: Goodness of fit index
AGFI: Adjusted goodness of fit
CFI: Comparative fit index
NFI: Normed fit index
IFI: Incremental fit index
TLI: Tucker-Lewis index
RMSEA: Root mean square error of approximation
RMR: Root mean residual
AVE: Average variance extracted
CR: Composite reliability
HTMT: Heterotrait-monotrait ratio of correlations
SE: Standard error
LLCI: Lower level of confidence interval
ULCI: Upper level of confidence interval

## References

[1] Hochschild AR (1979). Emotion work, feeling rules, and social structure. Am J Sociol.

[2] Abraham R (1998). Emotional dissonance in organizations: a conceptualization of consequences, mediators, and moderators. Leadersh Organ Dev J.

[3] Hülsheger UR, Schewe AF (2011). On the costs and benefits of emotional labor: a meta-analysis of three decades of research. J Occup Health Psychol.

[4] Grandey AA (2000). Emotion regulation in the workplace: a new way to conceptualize emotional labour. J Occup Health Psychol.

[5] Zhang H, Zhou ZE, Zhan Y, Liu C, Zhang L (2018). Surface acting, emotional exhaustion, and employee sabotage to customers: moderating roles of quality of social exchanges. Front Psychol.

[6] Grandey AA (2003). When the show must go on: surface acting and deep acting as determinants of emotional exhaustion and peer-rated service delivery. Acad Manage J.

[7] Jeon JH, Jo SJ. 2016. The impact of LMX on turnover intention of employees: Through the mediating effect of psychological empowerment and the moderating effect of informal learning. Korean J Hum Resour Dev Q. 2016;18(3):87–113; 10.18211/kjhrdq.2016.18.3.004.

[8] Allen JA, Diefendorff JM, Ma Y (2014). Differences in emotional labor across cultures: a comparison of Chinese and US service workers. J Bus Psychol.

[9] Humphrey NM (2021). Emotional labor and employee outcomes: a meta-analysis. Public Adm.

[10] Mastracci S, Adams I (2019). Is emotional labor easier in collectivist or individualist cultures? An East–West comparison. Public Pers Manag.

[11] Chen KY, Chang CW, Wang CH (2019). Frontline employees’ passion and emotional exhaustion: the mediating role of emotional labor strategies. Int J Hosp Manag.

[12] Lee SK, Shin HY, Lee HS (2016). The influence of police officer’s emotional labor on organizational effectiveness: focused on the mediating effect of job stress. J Police Sci.

[13] Kanasz T, Zielinska I (2017). Emotional labour of the Polish social workers: the study in sociology of emotions. Pol Sociol Rev.

[14] Kang MY, Han NY, Bae SW (2017). A study on the effects of emotional labour on counterproductive work behavior via burnout. Manag Inf Syst Rev.

[15] Park GW, Kim MS, Han Y (2014). Exploration of predictive variables of emotional labor in subordinate roles and verification of the relations model between emotional labor and burnout. Korean J Ind Organ Psychol.

[16] Yoon KS. The structural relationship among job performance, job crafting, person-job fit, informal learning, and job challenge and moderating effects of LMX and workload of office workers in large corporations [dissertation]. Seoul National University Graduate School; 2019.

[17] Lee JY (2009). Moderating effects of self-efficiency in the emotional labor and burnout process. J Hum Resour Manag Res.

[18] Kim TS, Hur C (2012). Effect of emotional labor on emotional exhaustion and job satisfaction: mainly on KTX train attendants. Korean J Bus Adm.

[19] Kwon HG, Park BG (2011). The effects of surface and deep acting of emotional labor on emotional dissonance and job attitudes. J Hum Resour Manag Res.

[20] Ga SM, Yoon DY, Han SH (2017). The effects of SME employee’s psychological capital on the organization commitment: the moderating effect of LMX. Korean Corp Manag Rev.

[21] Jeong J. The relationship among organizational adjustment, growth needs, informal learning, and leader-member exchange (LMX) of early career employees at small and medium-sized enterprises [dissertation]. Seoul National University Graduate School; 2019.

[22] Wu TJ, Yuan KS, Yen DC, Yeh CF (2023). The effects of JDC model on burnout and work engagement: a multiple interaction analysis. Eur Manag J.

[23] Jun SY (2015). A study on the influence of emotional labour on job satisfaction: focusing on the moderating effect of perceived organizational support. Korean Acad Association Bus Adm.

[24] Ashforth BE, Humphrey RH (1993). Emotional labor in service roles: the influence of identity. Acad Manage Rev.

[25] Rafaeli A, Sutton RI (1989). The expression of emotion in organizational life. Res Organ Behav.

[26] Morris JA, Feldman DC (1996). The dimensions, antecedents, and consequences of emotional labor. Acad Manage Rev.

[27] Yang S, Lee D (2019). The effects of emotional labor (surface acting, deep acting) and job burnout on job satisfaction among securities branch clerks: Moderated mediating effect of emotional intelligence. Korean J Ind Organ Psychol.

[28] Song HJ, Cho YJ (2016). Effects of emotional labor on burnout and satisfaction: focusing on social workers. Korean Public Adm Rev.

[29] Güler M, Ocak M, Köksal O (2023). The moderating role of surface and deep acting on the relationship between natural emotional labor and organizational commitment. Curr Psychol.

[30] Chang SCS, Chung A, Chen SY, Lin CY, Chen IH. Servant leadership and nurses’ deep acting: a moderated mediation model. Journal of Organizational Change Management, Vol. ahead-of-print No. ahead-of-print. 2023; 10.1108/JOCM-05-2023-0162.

[31] Hochschild AR (1983). Comment on Kemper’s social constructionist and positivist approaches to the sociology of emotions’. Am J Sociol.

[32] Grandey AA, Rupp D, Brice WN (2015). Emotional labour threatens decent work: a proposal to eradicate emotional display rules. J Organ Behav.

[33] McCance AS, Nye CD, Wang L, Jones KS, Chiu CY (2013). Alleviating the burden of emotional labor: the role of social sharing. J Manag.

[34] Kim JS, Han SH (2017). Exploring a mediating role of job burnout: a study of the relationship between emotional labor and job satisfaction in Korean street-level bureaucrats. J Korean Assoc Organ Stud.

[35] De Leersnyder J, Mesquita B, Kim H, Eom K, Choi H (2014). Emotional fit with culture: a predictor of individual differences in relational well-being. Emotion.

[36] Eid M, Diener E (2001). Norms for experiencing emotions in different cultures: Inter- and intranational differences. J Pers Soc Psychol.

[37] Guy ME, Newman MA, Mastracci SH (2014). Emotional labour: putting the service in Public Service.

[38] Gabriel AS, Diefendorff JM (2015). Emotional labour dynamics: a momentary approach. Acad Manage J.

[39] Hu X, Kaplan S (2015). Is feeling good good enough? Differentiating discrete positive emotions at work. J Organ Behav.

[40] Humphrey NM (2023). Emotional labor and employee outcomes: a meta-analysis. Public Adm.

[41] Mastracci S, Adams I (2023). Is emotional labor easier in collectivist or individualist cultures? An east–west comparison. Public Personnel Management.

[42] Nixon AE, Ceylan S, Nelson CE, Alabak M (2020). Emotional labour, collectivism and strain: a comparison of Turkish and US service employees. Work Stress.

[43] Park HN. A study on the burnout of public social workers: Focused on the mediating effect of perceived emotional labor experience [dissertation] Seoul National University Graduate School of Public Administration; 2009.

[44] Park SH (2015). Analysis of characteristic of emotional labor occupation using KNOW and its policy implication. Employ Trends Brief.

[45] Kosar R, Ahmed T, Naqvi SMMR. Impact of emotional labor strategies on employee burnout mediating role of emotional dissonance and moderating role of workplace social support. Economic and Social Development: Book of Proceedings; 2016. p. 144.

[46] Brotheridge CM, Grandey AA (2002). Emotional labor and burnout: comparing two perspectives of people work. J Vocat Behav.

[47] Einarsen S, Hoel H, Notelaers G (2009). Measuring exposure to bullying and harassment at work: validity, factor structure and psychometric properties of the negative acts Questionnaire-revised. Work Stress.

[48] Lee CK, Lee DJ (2012). An empirical study on relationships between organizational justice and employee’s attitude: investigating a moderation effect of job burnout. Korean J Bus Adm.

[49] Park HI, Nam SK, Yang EJ (2011). Relationships of burnout with job attitudes and turnover intention among koreans: a meta-analysis. Korean J Ind Organ Psychol.

[50] Azanza G, Moriano JA, Molero F, Lévy Mangin JPL (2015). The effects of authentic leadership on turnover intention. Leadersh Organ Dev J.

[51] Jensen JM, Patel PC, Messersmith JG (2013). High-performance work systems and job control: consequences for anxiety, role overload, and turnover intentions. J Manag.

[52] Schaufeli WB, Maslach C, Marek T (2017). Professional Burnout: recent developments in theory and research.

[53] Chan YH, Nadler SS, Hargis MB (2015). Attitudinal and behavioral outcomes of employees’ psychological empowerment: a structural equation modeling approach. J Organ Cult Commun Confl.

[54] Duffy RD, Dik BJ, Douglass RP, England JW, Velez BL (2018). Work as a calling: a theoretical model. J Couns Psychol.

[55] Scott BA, Barnes CM, Wagner DT (2012). Chameleonic or consistent? A multilevel investigation of emotional labor variability and self-monitoring. Acad Manage J.

[56] Gross JJ, Levenson RW (1997). Hiding feelings: the acute effects of inhibiting negative and positive emotion. J Abnorm Psychol.

[57] Bechtoldt MN, Welk C, Zapf D, Hartig J (2007). Main and moderating effects of self-control, organizational justice, and emotional labour on counterproductive behavior at work. Eur J Work Organ Psychol.

[58] Weiss HM, Cropanzano R (1996). Affective events theory. A theoretical discussion of the structure, causes and consequences of affective experiences at work. Res Organ Behav.

[59] Johns G (2010). Presenteeism in the workplace: a review and research agenda. J Organ Behav.

[60] Karatuna I, Gök S. 2014. A study analyzing the association between post-traumatic embitterment disorder and workplace bullying. J Workplace Behav Health. 2014;29(2):127–142; 10.1080/15555240.2014.898569.

[61] Ghasemy M, Erfanian M, Gaskin JE (2020). Affective events theory as a theoretical lens for improving the working environment of academics in developing economies. J Appl Res High Educ.

[62] Gerstner CR, Day DV (1997). Meta-analytic review of leader-member exchange theory: correlates and construct issues. J Appl Psychol.

[63] Graen GB, Uhl-Bien M (1995). Relationship-based approach to leadership: development of leader-member exchange (LMX) theory of leadership over 25 years: applying a multi-level multi-domain perspective. Leadersh Q.

[64] Diefendorff JM, Croyle MH, Gosserand RH (2005). The dimensionality and antecedents of emotional labour strategies. J Vocat Behav.

[65] Settoon RP, Bennett N, Liden RC (1996). Social exchange in organizations: perceived organizational support, leader-member exchange, and employee reciprocity. J Appl Psychol.

[66] Chou RJA, Robert SA (2008). Workplace support, role overload, and job satisfaction of direct care workers in assisted living. J Health Soc Behav.

[67] Kim SB (2022). The moderating effects of social support of the relationship between job-stress and burnout of elementary school teachers, teaching ADHD students in integrated school environment. J Spec Educ Rehabil Sci.

[68] Wang T, Long L, Zhang Y, He W (2019). A social exchange perspective of employee–organization relationships and employee unethical pro-organizational behavior: the moderating role of individual moral identity. J Bus Ethics.

[69] Swift M, Balkin DB, Matusik SF (2010). Goal orientations and the motivation to share knowledge. J Knowledg Manag.

[70] Wang H, Law KS, Hackett RD, Wang D, Chen ZX (2005). Leader-member exchange as a mediator of the relationship between transformational leadership and followers’ performance and organizational citizenship behavior. Acad Manage J.

[71] Cho HT, Yang JS (2018). How perceptions of organizational politics influence self-determined motivation: the mediating role of work mood. Asia Pac Manag Rev.

[72] Anand S, Vidyarthi P, Rolnicki S (2018). Leader-member exchange and organizational citizenship behaviors: contextual effects of leader power distance and group task interdependence. Leadersh Q.

[73] Eva N, Robin M, Sendjaya S, Van Dierendonck D, Liden RC (2019). Servant leadership: a systematic review and call for future research. Leadersh Q.

[74] Harris TB, Li N, Kirkman BL (2014). Leader-member exchange (LMX) in context: how LMX differentiation and LMX relational separation attenuate LMX’s influence on OCB and turnover intention. Leadersh Q.

[75] Wu TJ, Yuan KS, Yen DC (2023). Leader-member exchange, turnover intention and presenteeism–the moderated mediating effect of perceived organizational support. Curr Psychol.

[76] Chen Y, Wen Z, Peng J, Liu X (2016). Leader-follower congruence in loneliness, LMX and turnover intention. J Manag Psychol.

[77] Faul F, Erdfelder E, Lang AG, Buchner A (2007). G* power 3: a flexible statistical power analysis program for the social, behavioral, and biomedical sciences. Behav Res Methods.

[78] Maslach C, Jackson SE (1981). The measurement of experienced burnout. J Organ Behav.

[79] Kang HK. Analysis of the relationship between job stress characteristics and factors among special school teachers and general school teachers [dissertation]. Daegu University; 1996.

[80] Jang HI, Kim DY (2020). A study on the difference of emotional labor and burnout: focusing on teacher’s group, gender and career. J Learn Cent Curric Instr.

[81] Kim HJ (2014). A study on the emotional labor and job satisfaction of major supermarket sales clerk: by having social support as moderating variable. J Labor Stud.

[82] Landau J, Hammer TH (1986). Clerical employees’ perceptions of intraorganizational career opportunities. Acad Manage J.

[83] Hayes AF (2018). Partial, conditional, and moderated moderated mediation: quantification, inference, and interpretation. Commun Monogr.

[84] Yu JP. Concept and understanding of Professor Yu Jong-Pil’s Structural equation Model Rev. Edn. Hanna Rae Publishing; 2022.

[85] Podsakoff PM, Organ DW. (1986). Self-reports in organizational research: Problems and prospects. J Manag. 1986;12(4):531–544; 10.1177/014920638601200408.

[86] Fornell C, Larcker DF (1981). Evaluating structural equation models with unobservable variables and measurement error. J Mark Res.

[87] Hair JF, Hult GTM, Ringle CM, Sarstedt M. A Primer on Partial Least Squares Structural Equation Modeling (PLS-SEM) (3e). Thousand Oaks, CA: Sage; 2022.

[88] Zhao JL, Li XH, Shields J. 2019. Managing job burnout: The effects of emotion-regulation ability, emotional labour, and positive and negative affect at work. Int J Stress Manag. 2019;26(3):315–320; 10.1037/str0000101.

[89] West SG, Aiken LS, Krull JL. 1996. Experimental Personality Designs: Analyzing Categorical by Continuous Variable Interactions. J Pers. 1996;64(1):1–48. 10.1111/j.1467-6494.1996.tb00813.x 8656311

[90] Chau SL, Dahling JJ, Levy PE, Diefendorff JM (2009). A predictive study of emotional labour and turnover. J Organ Behav.

[91] Jeong KJ, Yoon HM (2016). Child-care teachers’ emotional labor, job stress, psychological well-being, and turnover intention. Korea J Child Care Educ.

[92] Seo JH (2012). The influence of emotional labor of elementary school sports instructors on organizational commitment and turnover intention. Korean J Elem Phys Educ.

[93] Shim CH, Kim YS (2013). The effects of hotel employees’ emotional labor on turnover intention focused on moderating effects of coping strategy. J Foodserv Manag.

[94] Shim JS, Jeong SE (2013). The effects of emotional labor and job burnout on customer orientation. J Bus Educ.

[95] Yin H, Huang S, Chen G (2019). The relationships between teachers’ emotional labor and their burnout and satisfaction: a meta-analytic review. Educ Res Rev.

[96] Im SH, Li MM, Lee YE (2021). The relationships between employees’ emotional labor, job burnout, job satisfaction and turnover intentions in Korean franchised restaurants. J Korean Soc Food Sci Nutr.

[97] Steinberg RJ, Figart DM (1990). Emotional labor since: the managed heart. Ann Am Acad Pol Soc Sci.

[98] Grandey AA, Fisk GM, Steiner DD (2005). Must service with a smile be stressful? The moderating role of personal control for American and French employees. J Appl Psychol.

[99] Diefendorff JM, Croyle MH, Gosserand RH (2005). The dimensionality and antecedents of emotional labor strategies. J Vocat Behav.

[100] Lee SW, Park SK (2019). The relationships between emotional labor, burnout, and job satisfaction among social workers: focusing on surface acting and deep acting. J Community Welf.

[101] Kammeyer-Mueller JD, Rubenstein AL, Long DM, Odio MA, Buckman BR (2013). A meta‐analytic structural model of dispositional affectivity and emotional labor. Pers Psychol.

[102] Tanaka HS, Ishiyama N. Effects of talent status and leader-member exchange on innovative work behaviour in talent management in Japan. Asia Pac Bus Rev. 2023;1–18. 10.1080/13602381.2023.2186623.

[103] Wu TY, Hu C (2013). Abusive supervision and subordinate emotional labor: the moderating role of openness personality. J Appl Soc Psychol.

[104] Grandey AA (2000). Emotional regulation in the workplace: a new way to conceptualize emotional labor. J Occup Health Psychol.

[105] Evans DC, Garcia DJ, Garcia DM, Baron RS. 2003. In the privacy of their own homes: Using the Internet to assess racial bias. Pers Soc Psychol Bull. 2003;29(2):273–284; 10.1177/0146167202239052. 10.1177/0146167202239052 15272954

