# Supplementary material for: A study on the structural relationship between emotional labor, job burnout, and turnover intention among office workers in Korea: the moderated mediating effect of leader-member exchange
Source: BMC Psychol. 2024 Jan 29;12:54. doi: 10.1186/s40359-024-01545-8 (PMC10826281; doi:10.1186/s40359-024-01545-8)
Supplement: Supplementary file 1 — Supplementary Material 1 [file 40359_2024_1545_MOESM1_ESM.pdf]

For use by Yiran Li only. Received from Mind Garden, Inc. on December 5, 2023

Permission for Yiran Li to administer 333 copies within three years of December 5, 2023

Maslach Burnout Inventory™

MBI Forms and Scoring Keys:

Human Services - MBI-HSS
Medical Personnel - MBI-HSS (MP)
Educators - MBI-ES
General - MBI-GS
Students - MBI-GS (S)

License to Administer

By Christina Maslach, Susan E. Jackson, Michael P. Leiter,
Wilmar B. Schaufeli & Richard L. Schwab

Published by Mind Garden, Inc.
www.mindgarden.com

Important Note to Licensee

It is your legal responsibility to compensate the copyright holder of this work — via payment to Mind Garden — for reproduction or administration in any physical or digital medium, including online survey, handheld survey devices, etc.

You agree to track the number of reproductions or administrations, and to compensate Mind Garden for any usage in excess of the quantity purchased.

This license is valid for three years from the date of purchase.

This instrument, and any use thereof, is covered by U.S. and international copyright laws. For any further use or reproduction of the instrument, in whole or in part, contact Mind Garden, Inc.

MBI-Human Services Survey: Copyright ©1981 Christina Maslach & Susan E. Jackson.
MBI-Human Services Survey for Medical Personnel: Copyright ©1981, 2016 Christina Maslach & Susan E. Jackson.
MBI-Educators Survey: Copyright ©1986 Christina Maslach, Susan E. Jackson & Richard L. Schwab.
MBI-General Survey: Copyright ©1996 Wilmar B. Schaufeli, Michael P. Leiter, Christina Maslach & Susan E. Jackson.
MBI-General Survey for Students: Copyright ©1996, 2016 Wilmar B. Schaufeli, Michael P. Leiter, Christina Maslach & Susan E. Jackson.
All rights reserved in all media. Published by Mind Garden, Inc., www.mindgarden.com

For use by Yiran Li only. Received from Mind Garden, Inc. on December 5, 2023

Permission Letter

www.mindgarden.com

To Whom It May Concern,

The above-named person has made a license purchase from Mind Garden, Inc. and has permission to administer the following copyrighted instrument up to that quantity purchased:

Maslach Burnout Inventory forms: Human Services Survey, Human Services Survey for Medical Personnel, Educators Survey, General Survey, or General Survey for Students.

The license holder has permission to administer the complete instrument in their research, however, only three sample items from this instrument as specified below may be included in the research write-up, thesis, or dissertation. Any other use must receive prior written permission from Mind Garden. The entire instrument form may not be included or reproduced at any time in any other published material. Please understand that disclosing more than we have authorized will compromise the integrity and value of the test.

Citation of the instrument must include the applicable copyright statement listed below.
Sample Items:

MBI - Human Services Survey - MBI-HSS:
I feel emotionally drained from my work.
I have accomplished many worthwhile things in this job.
I don’t really care what happens to some recipients.

Copyright ©1981 Christina Maslach & Susan E. Jackson. All rights reserved in all media.
Published by Mind Garden, Inc., www.mindgarden.com

MBI - Human Services Survey for Medical Personnel - MBI-HSS (MP):
I feel emotionally drained from my work.
I have accomplished many worthwhile things in this job.
I don’t really care what happens to some patients.

Copyright ©1981, 2016 by Christina Maslach & Susan E. Jackson. All rights reserved in all media.
Published by Mind Garden, Inc., www.mindgarden.com

MBI - Educators Survey - MBI-ES:
I feel emotionally drained from my work.
I have accomplished many worthwhile things in this job.
I don’t really care what happens to some students.

Copyright ©1986 Christina Maslach, Susan E. Jackson & Richard L. Schwab. All rights reserved in all media. Published by Mind Garden, Inc., www.mindgarden.com

Cont’d on next page

For use by Yiran Li only. Received from Mind Garden, Inc. on December 5, 2023

MBI - General Survey - MBI-GS:

I feel emotionally drained from my work.
In my opinion, I am good at my job.
I doubt the significance of my work.

Copyright ©1996 Wilmar B. Schaufeli, Michael P. Leiter, Christina Maslach & Susan E. Jackson. All rights reserved in all media. Published by Mind Garden, Inc., www.mindgarden.com

MBI - General Survey for Students - MBI-GS (S):

I feel emotionally drained by my studies.
In my opinion, I am a good student.
I doubt the significance of my studies.

Copyright ©1996, 2016 Wilmar B. Schaufeli, Michael P. Leiter, Christina Maslach & Susan E. Jackson. All rights reserved in all media. Published by Mind Garden, Inc., www.mindgarden.com

Sincerely,

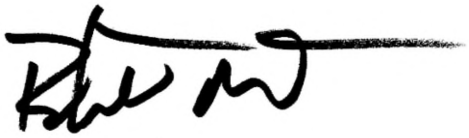

Robert Most
Mind Garden, Inc.
www.mindgarden.com

For use by Yiran Li only. Received from Mind Garden, Inc. on December 5, 2023

Conditions for Online Use

You agree to:

- Administer this Mind Garden instrument for research purposes only.
- Control access to the instrument. The instrument may not be made available via the open web, nor sent in the message body or as an attachment to survey participants.
- Avoid using copyrighted items from this instrument on the survey landing page. You should put other items on this landing page, e.g., demographics or informed consent.
- Put the instrument copyright statement on every page containing questions/items from this instrument. The copyright statement is provided in the enclosed permission letter.
- Track your license use. Each time a participant opens the survey and has access to the copyrighted items is considered one administration.
- Compensate Mind Garden, Inc. for each administration.
- Purchase a license for any additional administrations over the initial purchase quantity, or close the survey.
- Remove this online survey at the conclusion of your data collection and you will personally confirm that it cannot be accessed.

Caution: If you do not require a unique login for each respondent, the survey method you use may elicit a large number of responses to your survey. You are responsible for compensating Mind Garden for every administration, regardless of circumstances.

Distributing an entire instrument in either the text of an email or as an email attachment is strictly prohibited.

For use by Yiran Li only. Received from Mind Garden, Inc. on December 5, 2023

For Dissertation and Thesis Appendices

This license permits you to administer the complete instrument in your research, however, only the three pre-authorized sample items from this instrument as provided by Mind Garden may be included in the research write-up, thesis, dissertation, or appendices, and only when accompanied by the instrument copyright statement. Detailed information on these requirements is provided in the enclosed permission letter.

For Results Reporting and Publications

This research license is for data collection, and it permits you to collect and disclose item scores, scale scores, and scores statistics (group average, group standard deviation, T-scores, etc.). You may not include the complete instrument in results reporting or publications -- you may include only the three pre-authorized sample items and with copyright language and attribution: see the enclosed permission letter.

This license is for research only and not for providing individual feedback to survey participants. Please understand that disclosing more than we have authorized will compromise the integrity and value of the test.

Note: the list above illustrates some permitted and prohibited uses of the instrument and is not meant to be comprehensive.

Translations

Translations are available free of charge with license purchase. Contact info@mindgarden.com to request translations. If you would like to make a translation, please complete the Translation Application, available at https://www.mindgarden.com/mind-garden-forms/61-translation-application.html

All Other Reproductions

For any other reproductions or permissions, please contact info@mindgarden.com

For use by Yiran Li only. Received from Mind Garden, Inc. on December 5, 2023

Note to Survey Administrators

Avoid Sensitization to Burnout. People have widely varying beliefs about burnout. To minimize the reactive effect of such personal beliefs or expectations, it is important that respondents be unaware that the MBI is a burnout measure and that they not be sensitized to the general issue of burnout. For this reason, the labels used on the survey do not include the word “burnout”. You will often see survey administrators use one of the following acceptable terms or similar wording on the survey or referring to it:

- MBI Assessment
- Wellness Survey
- Job Attitudes Assessment
- Employee Well-Being Survey

The scale should be presented as a survey of job-related attitudes and not be linked to burnout in any way. Of course, once the measure has been administered to all respondents, then an explanation about burnout and the use of the MBI to assess it is appropriate.

For use by Yiran Li only. Received from Mind Garden, Inc. on December 5, 2023

Human Services Survey

Christina Maslach & Susan E. Jackson

The purpose of this survey is to discover how various people working in human services or the helping professions view their job and the people with whom they work closely.

Because people in a wide variety of occupations will answer this survey, it uses the term *recipients* to refer to the people for whom you provide your service, care, treatment, or instruction. When answering this survey please think of these people as recipients of the service you provide, even though you may use another term in your work.

Instructions: On the following page are 22 statements of job-related feelings. Please read each statement carefully and decide if you ever feel this way about *your* job. If you have *never* had this feeling, write the number "0" (zero) in the space before the statement. If you have had this feeling, indicate *how often* you feel it by writing the number (from 1 to 6) that best describes how frequently you feel that way. An example is shown below.

Example:

**How often:**	**0**	**1**	**2**	**3**	**4**	**5**	**6**
	Never	A few times a year or less	Once a month or less	A few times a month	Once a week	A few times a week	Every day

**How often 0-6**	**Statement:**
1. _________	I feel depressed at work.

If you never feel depressed at work, you would write the number "0" (zero) under the heading "How often." If you rarely feel depressed at work (a few times a year or less), you would write the number "1." If your feelings of depression are fairly frequent (a few times a week but not daily), you would write the number "5."

MBI - Human Services Survey - MBI-HSS: Copyright ©1981 Christina Maslach & Susan E. Jackson.
All rights reserved in all media. Published by Mind Garden, Inc., www.mindgarden.com

For use by Yiran Li only. Received from Mind Garden, Inc. on December 5, 2023

MBI Human Services Survey

How often:	0	1	2	3	4	5	6
	Never	A few times a year or less	Once a month or less	A few times a month	Once a week	A few times a week	Every day

How often 0-6	Statements:
1. _________	I feel emotionally drained from my work.
2. _________	I feel used up at the end of the workday.
3. _________	I feel fatigued when I get up in the morning and have to face another day on the job.
4. _________	I can easily understand how my recipients feel about things.
5. _________	I feel I treat some recipients as if they were impersonal objects.
6. _________	Working with people all day is really a strain for me.
7. _________	I deal very effectively with the problems of my recipients.
8. _________	I feel burned out from my work.
9. _________	I feel I'm positively influencing other people's lives through my work.
10. _________	I've become more callous toward people since I took this job.
11. _________	I worry that this job is hardening me emotionally.
12. _________	I feel very energetic.
13. _________	I feel frustrated by my job.
14. _________	I feel I'm working too hard on my job.
15. _________	I don't really care what happens to some recipients.
16. _________	Working with people directly puts too much stress on me.
17. _________	I can easily create a relaxed atmosphere with my recipients.
18. _________	I feel exhilarated after working closely with my recipients.
19. _________	I have accomplished many worthwhile things in this job.
20. _________	I feel like I'm at the end of my rope.
21. _________	In my work, I deal with emotional problems very calmly.
22. _________	I feel recipients blame me for some of their problems.

(Administrative use only)

EE Total score: ________ DP Total score: ________ PA Total score: ________

EE Average score: ______ DP Average score: ______ PA Average score: ______

MBI - Human Services Survey - MBI-HSS: Copyright ©1981 Christina Maslach & Susan E. Jackson.
All rights reserved in all media. Published by Mind Garden, Inc., www.mindgarden.com

For use by Yiran Li only. Received from Mind Garden, Inc. on December 5, 2023

MBI Human Services Survey for Medical Personnel

Christina Maslach & Susan E. Jackson

The purpose of this survey is to discover how various people in the human services or the helping professions view their job and the people with whom they work closely.

Instructions: On the following page are 22 statements of job-related feelings. Please read each statement carefully and decide if you ever feel this way about *your* job. If you have *never* had this feeling, write the number “0” (zero) in the space before the statement. If you have had this feeling, indicate *how often* you feel it by writing the number (from 1 to 6) that best describes how frequently you feel that way. An example is shown below.

Example:

**How often:**	**0**	**1**	**2**	**3**	**4**	**5**	**6**
	Never	A few times a year or less	Once a month or less	A few times a month	Once a week	A few times a week	Every day

**How often 0-6**	**Statement:**
1. _________	I feel depressed at work.

If you never feel depressed at work, you would write the number “0” (zero) under the heading “How often.” If you rarely feel depressed at work (a few times a year or less), you would write the number “1.” If your feelings of depression are fairly frequent (a few times a week but not daily), you would write the number “5.”

MBI - Human Services Survey for Medical Personnel - MBI-HSS (MP): Copyright ©1981, 2016 Christina Maslach & Susan E. Jackson. All rights reserved in all media. Published by Mind Garden, Inc., www.mindgarden.com

For use by Yiran Li only. Received from Mind Garden, Inc. on December 5, 2023

MBI Human Services Survey for Medical Personnel

How often:	0	1	2	3	4	5	6
	Never	A few times a year or less	Once a month or less	A few times a month	Once a week	A few times a week	Every day

How often 0-6	Statements:
1. _________	I feel emotionally drained from my work.
2. _________	I feel used up at the end of the workday.
3. _________	I feel fatigued when I get up in the morning and have to face another day on the job.
4. _________	I can easily understand how my patients feel about things.
5. _________	I feel I treat some patients as if they were impersonal objects.
6. _________	Working with people all day is really a strain for me.
7. _________	I deal very effectively with the problems of my patients.
8. _________	I feel burned out from my work.
9. _________	I feel I'm positively influencing other people's lives through my work.
10. _________	I've become more callous toward people since I took this job.
11. _________	I worry that this job is hardening me emotionally.
12. _________	I feel very energetic.
13. _________	I feel frustrated by my job.
14. _________	I feel I'm working too hard on my job.
15. _________	I don't really care what happens to some patients.
16. _________	Working with people directly puts too much stress on me.
17. _________	I can easily create a relaxed atmosphere with my patients.
18. _________	I feel exhilarated after working closely with my patients.
19. _________	I have accomplished many worthwhile things in this job.
20. _________	I feel like I'm at the end of my rope.
21. _________	In my work, I deal with emotional problems very calmly.
22. _________	I feel patients blame me for some of their problems.

(Administrative use only)

EE Total score: _________	DP Total score: _________	PA Total score: __________
EE Average score: ______	DP Average score: _______	PA Average score: _______

MBI - Human Services Survey for Medical Personnel - MBI-HSS (MP): Copyright ©1981, 2016 Christina Maslach & Susan E. Jackson. All rights reserved in all media. Published by Mind Garden, Inc., www.mindgarden.com

For use by Yiran Li only. Received from Mind Garden, Inc. on December 5, 2023

MBI for Educators Survey

Christina Maslach, Susan E. Jackson & Richard L. Schwab

The purpose of this survey is to discover how educators view their job and the people with whom they work closely.

Instructions: On the following page are 22 statements of job-related feelings. Please read each statement carefully and decide if you ever feel this way about *your* job. If you have *never* had this feeling, write the number "0" (zero) in the space before the statement. If you have had this feeling, indicate *how often* you feel it by writing the number (from 1 to 6) that best describes how frequently you feel that way. An example is shown below.

How often:	0	1	2	3	4	5	6
	Never	A few times a year or less	Once a month or less	A few times a month	Once a week	A few times a week	Every day

Example:

How often 0-6	Statement:
1. _________	I feel depressed at work.

If you never feel depressed at work, you would write the number "0" (zero) under the heading "How often." If you rarely feel depressed at work (a few times a year or less), you would write the number "1." If your feelings of depression are fairly frequent (a few times a week but not daily), you would write the number "5."

MBI - Educators Survey - MBI-ES: Copyright ©1986 Christina Maslach, Susan E. Jackson & Richard L. Schwab. All rights reserved in all media. Published by Mind Garden, Inc., www.mindgarden.com

For use by Yiran Li only. Received from Mind Garden, Inc. on December 5, 2023

MBI for Educators Survey

How often:	0	1	2	3	4	5	6
	Never	A few times a year or less	Once a month or less	A few times a month	Once a week	A few times a week	Every day

How often 0-6	Statements:
1. _________	I feel emotionally drained from my work.
2. _________	I feel used up at the end of the workday.
3. _________	I feel fatigued when I get up in the morning and have to face another day on the job.
4. _________	I can easily understand how my students feel about things.
5. _________	I feel I treat some students as if they were impersonal objects.
6. _________	Working with people all day is really a strain for me.
7. _________	I deal very effectively with the problems of my students.
8. _________	I feel burned out from my work.
9. _________	I feel I'm positively influencing other people's lives through my work.
10. _________	I've become more callous toward people since I took this job.
11. _________	I worry that this job is hardening me emotionally.
12. _________	I feel very energetic.
13. _________	I feel frustrated by my job.
14. _________	I feel I'm working too hard on my job.
15. _________	I don't really care what happens to some students.
16. _________	Working with people directly puts too much stress on me.
17. _________	I can easily create a relaxed atmosphere with my students.
18. _________	I feel exhilarated after working closely with my students.
19. _________	I have accomplished many worthwhile things in this job.
20. _________	I feel like I'm at the end of my rope.
21. _________	In my work, I deal with emotional problems very calmly.
22. _________	I feel students blame me for some of their problems.

(Administrative use only)

EE Total score: _________ DP Total score: _________ PA Total score: _________

EE Average score: ______ DP Average score: ______ PA Average score: _______

MBI - Educators Survey - MBI-ES: Copyright ©1986 Christina Maslach, Susan E. Jackson & Richard L. Schwab. All rights reserved in all media. Published by Mind Garden, Inc., www.mindgarden.com

For use by Yiran Li only. Received from Mind Garden, Inc. on December 5, 2023

MBI – Human Services, Medical Personnel, and Educators Scoring Key
Emotional Exhaustion (EE) Subscale

Directions: Line up this scoring key with the MBI survey form. Sum the survey responses on EE items # 1, 2, 3, 6, 8, 13, 14, 16, and 20 that correspond to the unshaded areas on this scoring key. Enter this EE total score on the survey form. Divide the EE total score by the number of answered EE items for an EE average score. Research usually reports the average score.

How Often 0-6
1. _________
2. _________
3. _________
4. _________
5. _________
6. _________
7. _________
8. _________
9. _________
10. _________
11. _________
12. _________
13. _________
14. _________
15. _________
16. _________
17. _________
18. _________
19. _________
20. _________
21. _________
22. _________

MBI - Human Services Survey - MBI-HSS: Copyright ©1981 Christina Maslach & Susan E. Jackson.

MBI - Human Services Survey for Medical Personnel - MBI-HSS (MP): Copyright ©1981, 2016 by Christina Maslach & Susan E. Jackson.

MBI - Educators Survey - MBI-ES: Copyright ©1986 Christina Maslach, Susan E. Jackson & Richard L. Schwab. All rights reserved in all media. Published by Mind Garden, Inc., www.mindgarden.com

For use by Yiran Li only. Received from Mind Garden, Inc. on December 5, 2023

MBI – Human Services, Medical Personnel, and Educators Scoring Key Depersonalization (DP) Subscale

Directions: Line up this scoring key with the MBI survey form. Sum the survey responses on DP items # 5, 10, 11, 15, and 22 that correspond to the unshaded areas on this scoring key. Enter this DP total score on the survey form. Divide the DP total score by the number of answered DP items for a DP average score. Research usually reports the average score.

How Often 0-6
1. _________
2. _________
3. _________
4. _________
5. _________
6. _________
7. _________
8. _________
9. _________
10. _________
11. _________
12. _________
13. _________
14. _________
15. _________
16. _________
17. _________
18. _________
19. _________
20. _________
21. _________
22. _________

MBI – Human Services Survey - MBI-HSS: Copyright ©1981 Christina Maslach & Susan E. Jackson.

MBI - Human Services Survey for Medical Personnel - MBI-HSS (MP): Copyright ©1981, 2016 by Christina Maslach & Susan E. Jackson.

MBI - Educators Survey - MBI-ES: Copyright ©1986 Christina Maslach, Susan E. Jackson & Richard L. Schwab. All rights reserved in all media. Published by Mind Garden, Inc., www.mindgarden.com

For use by Yiran Li only. Received from Mind Garden, Inc. on December 5, 2023

MBI – Human Services, Medical Personnel, and Educators Scoring Key Personal Accomplishment (PA) Subscale

Directions: Line up this scoring key with the MBI survey form. Sum the survey responses on PA items # 4, 7, 9, 12, 17, 18, 19, and 21 that correspond to the unshaded areas on this scoring key. Enter this PA total score on the survey form. Divide the PA total score by the number of answered PA items for a PA average score. Research usually reports the average score.

How Often 0-6
1. _________
2. _________
3. _________
4. _________
5. _________
6. _________
7. _________
8. _________
9. _________
10. _________
11. _________
12. _________
13. _________
14. _________
15. _________
16. _________
17. _________
18. _________
19. _________
20. _________
21. _________
22. _________

MBI – Human Services Survey - MBI-HSS: Copyright ©1981 Christina Maslach & Susan E. Jackson.

MBI - Human Services Survey for Medical Personnel - MBI-HSS (MP): Copyright ©1981, 2016 by Christina Maslach & Susan E. Jackson.

MBI - Educators Survey - MBI-ES: Copyright ©1986 Christina Maslach, Susan E. Jackson & Richard L. Schwab. All rights reserved in all media. Published by Mind Garden, Inc., www.mindgarden.com

For use by Yiran Li only. Received from Mind Garden, Inc. on December 5, 2023

MBI - General Survey

Wilmar B. Schaufeli, Michael P. Leiter, Christina Maslach & Susan E. Jackson

The purpose of this survey is to discover how staff members view their job, and their reactions to their work.

Instructions: On the following page are 16 statements of job-related feelings. Please read each statement carefully and decide if you ever feel this way about *your* job. If you have *never* had this feeling, write the number “0” (zero) in the space before the statement. If you have had this feeling, indicate *how often* you feel it by writing the number (from 1 to 6) that best describes how frequently you feel that way. An example is shown below.

Example:

**How often:**	**0**	**1**	**2**	**3**	**4**	**5**	**6**
	Never	A few times a year or less	Once a month or less	A few times a month	Once a week	A few times a week	Every day

**How often 0-6**	**Statement:**
1. _________	I feel depressed at work.

If you never feel depressed at work, you would write the number “0” (zero) under the heading “How often.” If you rarely feel depressed at work (a few times a year or less), you would write the number “1.” If your feelings of depression are fairly frequent (a few times a week but not daily), you would write the number “5.”

MBI - General Survey - MBI-GS: Copyright ©1996 Wilmar B. Schaufeli, Michael P. Leiter, Christina Maslach & Susan E. Jackson. All rights reserved in all media. Published by Mind Garden, Inc., www.mindgarden.com

For use by Yiran Li only. Received from Mind Garden, Inc. on December 5, 2023

MBI - General Survey

How often:	0	1	2	3	4	5	6
	Never	A few times a year or less	Once a month or less	A few times a month	Once a week	A few times a week	Every day

How often 0-6	Statements:
1. ________	I feel emotionally drained from my work.
2. ________	I feel used up at the end of the workday.
3. ________	I feel tired when I get up in the morning and have to face another day on the job.
4. ________	Working all day is really a strain for me.
5. ________	I can effectively solve the problems that arise in my work.
6. ________	I feel burned out from my work.
7. ________	I feel I am making an effective contribution to what this organization does.
8. ________	I have become less interested in my work since I started this job.
9. ________	I have become less enthusiastic about my work.
10. ________	In my opinion, I am good at my job.
11. ________	I feel exhilarated when I accomplish something at work.
12. ________	I have accomplished many worthwhile things in this job.
13. ________	I just want to do my job and not be bothered.
14. ________	I have become more cynical about whether my work contributes anything.
15. ________	I doubt the significance of my work.
16. ________	At my work, I feel confident that I am effective at getting things done.

(Administrative use only)

EX Total score: ________	CY Total score: ________	PE Total score: _________
EX Average score: ______	CY Average score: ______	PE Average score:_______

MBI - General Survey - MBI-GS: Copyright ©1996 Wilmar B. Schaufeli, Michael P. Leiter, Christina Maslach & Susan E. Jackson. All rights reserved in all media. Published by Mind Garden, Inc., www.mindgarden.com

For use by Yiran Li only. Received from Mind Garden, Inc. on December 5, 2023

MBI - General Survey for Students

Wilmar B. Schaufeli, Michael P. Leiter, Christina Maslach & Susan E. Jackson

The purpose of this survey is to discover how university students view their studies, and their reactions to their academic work.

Instructions: On the following page are 16 statements of university-related feelings. Please read each statement carefully and decide if you ever feel this way about *your* academic work. If you have *never* had this feeling, write the number “0” (zero) in the space before the statement. If you have had this feeling, indicate *how often* you feel it by writing the number (from 1 to 6) that best describes how frequently you feel that way. An example is shown below.

Example:

**How often:**	**0**	**1**	**2**	**3**	**4**	**5**	**6**
	Never	A few times a year or less	Once a month or less	A few times a month	Once a week	A few times a week	Every day

**How often 0-6**	**Statement:**
1. _________	I feel depressed by my studies.

If you never feel depressed by your studies, you would write the number “0” (zero) under the heading “How often.” If you rarely feel depressed by your studies, (a few times a year or less), you would write the number “1.” If your feelings of being depressed by your studies are fairly frequent (a few times a week but not daily), you would write the number “5.”

MBI - General Survey for Students – MBI-GS (S): Copyright ©1996, 2016 Wilmar B. Schaufeli, Michael P. Leiter, Christina Maslach & Susan E. Jackson.   All rights reserved in all media. Published by Mind Garden, Inc. www.mindgarden.com.

For use by Yiran Li only. Received from Mind Garden, Inc. on December 5, 2023

MBI - General Survey for Students

**How often:**	**0**	**1**	**2**	**3**	**4**	**5**	**6**
	Never	A few times a year or less	Once a month or less	A few times a month	Once a week	A few times a week	Every day

How Often 0-6 **Statements:**

1. ______ I feel emotionally drained by my studies.
2. ______ I feel used up at the end of the day at the university.
3. ______ I feel tired when I get up in the morning and have to face another day at the university.
4. ______ Attending classes all day is really a strain for me.
5. ______ I can effectively solve the problems that arise in my studies.
6. ______ I feel burned out from my studies.
7. ______ I feel I am making an effective contribution in class.
8. ______ I have become less interested in my studies since my enrollment.
9. ______ I have become less enthusiastic about my studies.
10. ______ In my opinion, I am a good student.
11. ______ I feel exhilarated when I accomplish something at the university.
12. ______ I have accomplished many worthwhile things in my studies.
13. ______ I just want to get my work done and not be bothered.
14. ______ I have become more cynical about whether my university work contributes anything.
15. ______ I doubt the significance of my studies.
16. ______ While working at the university, I feel confident that I am effective at getting things done.

(Administrative use only)

EX Total score: ________ CY Total score: ________ PE Total score: _________

EX Average score: ______ CY Average score: ______ PE Average score: ______

MBI - General Survey for Students – MBI-GS (S): Copyright ©1996, 2016 Wilmar B. Schaufeli, Michael P. Leiter, Christina Maslach & Susan E. Jackson. All rights reserved in all media. Published by Mind Garden, Inc. www.mindgarden.com.

For use by Yiran Li only. Received from Mind Garden, Inc. on December 5, 2023

MBI – General Survey and General Survey for Students Scoring Key Exhaustion (EX) Subscale

Directions: Line up this scoring key with the MBI survey form. Sum the survey responses on EX items # 1, 2, 3, 4, and 6 that correspond to the unshaded areas on this scoring key. Enter this EX total score on the survey form. Divide the EX total score by the number of answered EX items for an EX average score. Research usually reports the average score.

**How Often 0-6**
1. _________
2. _________
3. _________
4. _________
5. _________
6. _________
7. _________
8. _________
9. _________
10. _________
11. _________
12. _________
13. _________
14. _________
15. _________
16. _________

MBI - General Survey – MBI-GS: Copyright ©1996 Wilmar B. Schaufeli, Michael P. Leiter, Christina Maslach & Susan E. Jackson.

MBI - General Survey for Students – MBI-GS (S): Copyright ©1996, 2016 Wilmar B. Schaufeli, Michael P. Leiter, Christina Maslach & Susan E. Jackson.

All rights reserved in all media. Published by Mind Garden, Inc. www.mindgarden.com.

For use by Yiran Li only. Received from Mind Garden, Inc. on December 5, 2023

MBI – General Survey and General Survey for Students Scoring Key Cynicism (CY) Subscale

Directions: Line up this scoring key with the MBI survey form. Sum the survey responses on CY items # 8, 9, 13, 14, and 15 that correspond to the unshaded areas on this scoring key. Enter this CY total score on the survey form. Divide the CY total score by the number of answered CY items for a CY average score. Research usually reports the average score.

How Often 0-6
1. _________
2. _________
3. _________
4. _________
5. _________
6. _________
7. _________
8. _________
9. _________
10. _________
11. _________
12. _________
13. _________
14. _________
15. _________
16. _________

MBI - General Survey – MBI-GS: Copyright ©1996 Wilmar B. Schaufeli, Michael P. Leiter, Christina Maslach & Susan E. Jackson.

MBI - General Survey for Students – MBI-GS (S): Copyright ©1996, 2016 Wilmar B. Schaufeli, Michael P. Leiter, Christina Maslach & Susan E. Jackson.

All rights reserved in all media. Published by Mind Garden, Inc. www.mindgarden.com.

For use by Yiran Li only. Received from Mind Garden, Inc. on December 5, 2023

MBI – General Survey and General Survey for Students Scoring Key Professional Efficacy (PE) Subscale

Directions: Line up this scoring key with the MBI survey form. Sum the survey responses on PE items # 5, 7, 10, 11, 12, and 16 that correspond to the unshaded areas on this scoring key. Enter this PE total score on the survey form. Divide the PE total score by the number of answered PE items for a PE average score. Research usually reports the average score.

How Often 0-6	
1.	_________
2.	_________
3.	_________
4.	_________
5.	_________
6.	_________
7.	_________
8.	_________
9.	_________
10.	_________
11.	_________
12.	_________
13.	_________
14.	_________
15.	_________
16.	_________

MBI - General Survey – MBI-GS: Copyright ©1996 Wilmar B. Schaufeli, Michael P. Leiter, Christina Maslach & Susan E. Jackson.

MBI - General Survey for Students – MBI-GS (S): Copyright ©1996, 2016 Wilmar B. Schaufeli, Michael P. Leiter, Christina Maslach & Susan E. Jackson.

All rights reserved in all media. Published by Mind Garden, Inc. www.mindgarden.com.
